# Fiber reinforced hydrated networks recapitulate the poroelastic mechanics of articular cartilage

**DOI:** 10.1016/j.actbio.2023.06.015

**Published:** 2023-06-17

**Authors:** A.C. Moore, M.G. Hennessy, L.P. Nogueira, S.J. Franks, M. Taffetani, H. Seong, Y.K. Kang, W.S. Tan, G. Miklosic, R. El Laham, K. Zhou, L. Zharova, J.R. King, B. Wagner, H.J. Haugen, A. Münch, M.M. Stevens

**Affiliations:** a Department of Materials, Department of Bioengineering and Institute of Biomedical Engineering, Imperial College London, London SW7 2AZ, UK; b Mathematical Institute, University of Oxford, Oxford OX2 6GG, UK; c Department of Engineering Mathematics, University of Bristol, Bristol BS8 1TW, UK; d Department of Biomaterials, Institute of Clinical Dentistry, University of Oslo, Oslo NO-0316, Norway; e School of Mathematical Sciences, University of Nottingham, Nottingham NG7 2RD, UK; f Weierstrass Institute for Applied Analysis and Stochastics, Berlin D-10117, Germany; g Oral Research Laboratory, Institute of Clinical Dentistry, University of Oslo, Oslo NO-0316, Norway

**Keywords:** Biphasic mechanics, Poroelastic mechanics, Engineered cartilage, Electrospinning, Interpenetrating network, Soft composite, Multiphasic mechanics

## Abstract

The role of poroelasticity on the functional performance of articular cartilage has been established in the scientific literature since the 1960s. Despite the extensive knowledge on this topic there remain few attempts to design for poroelasticity and to our knowledge no demonstration of an engineered poroelastic material that approaches the physiological performance. In this paper, we report on the development of an engineered material that begins to approach physiological poroelasticity. We quantify poroelasticity using the fluid load fraction, apply mixture theory to model the material system, and determine cytocompatibility using primary human mesenchymal stem cells. The design approach is based on a fiber reinforced hydrated network and uses routine fabrication methods (electrohydrodynamic deposition) and materials (poly[ε-caprolactone] and gelatin) to develop the engineered poroelastic material. This composite material achieved a mean peak fluid load fraction of 68%, displayed consistency with mixture theory, and demonstrated cytocompatibility. This work creates a foundation for designing poroelastic cartilage implants and developing scaffold systems to study chondrocyte mechanobiology and tissue engineering.

## 1 Introduction

Articular cartilage is the load-bearing and lubricating material of mammalian joints. Despite an equilibrium compressive modulus between 0.2 and 0.5 MPa [1], cartilage supports contact stresses that regularly range from 0.5 to 5 MPa and occasionally exceed 15 MPa [2]. Cartilage performs this mechanical feat of supporting compressive stresses in excess of its compressive modulus by transferring load from the solid matrix to the fluid through the development of interstitial fluid pressure (IFP) which is balanced by the stiff collagen network. The following example demonstrates the significance of IFP. *In vivo* measurements of the human knee have shown that daily activities (walking, standing, knee bends) produce strains on the order of 5% [3–6]. Under these conditions, the stress carried by the solid phase (modulus × strain) is ~0.025 MPa. Given a moderate contact stress of 1 MPa [7], the remaining compressive stress (0.975 MPa or 97.5% of the load) must be supported by IFP, which stiffens the contact, shields the solid matrix, and lubricates the sliding interface [8].

The high tensile stiffness collagen matrix of articular cartilage provides the necessary reaction force to build physiological IFP [9,10], enabling it to exceed the compressive modulus of the solid matrix. Without a reinforcing phase such as collagen, a linear isotropic poroelastic material is theoretically limited to supporting 33% (unconfined compression [9,11,12]) or 50% (spherical indentation [13]) of the applied load through IFP. In the case of confined compression, the upper bound is 100% due to the rigid walls of the confining chamber. Healthy articular cartilage has been shown to support 85 to 99% of the applied load by IFP [9,14–17].

To prolong this IFP requires an enmeshed hydrated network that resists fluid exudation [18,19]. Without this network, IFP would rapidly decay like wringing-out a sponge. To achieve this, cartilage employs proteoglycans which are macro biomolecules, on the order of MDa, decorated with glycosaminoglycans [18,20]. These glycosaminoglycans have a high affinity for water and carry a net negative charge that further increases the osmotic stress [19,21].

Poroelastic theory is one constitutive framework used to describe the physics underlying IFP [22]. There have been many different frameworks over the last 7 decades to describe the poroelastic nature of articular cartilage [23] (*e.g*., biphasic, triphasic, or multiphasic theory [24,25], consolidation theory [22,26], boosted lubrication [27], and weeping lubrication [28]). While differences exist between the modeling approaches, they all describe IFP, and for the purposes of this work we use the term poroelastic to generalize this concept. The extensive experimental and theoretical literature on this topic has identified two fundamental requirements for cartilage-like poroelasticity: 1) a water-swollen network and 2) a reinforcing matrix with high tensile-to-compressive stiffness (*i.e.,)*fibrous matrix).

Swollen polymeric networks (*e.g*., polyethylene glycol, alginate, agarose, fibrin, collagen, hyaluronic acid, and polyacrylamide) have long been investigated for cartilage applications due to their high water content and low friction; however, these materials are almost universally limited by their load carrying capacity [29–31]. In recent years, several groups have sought to enhance the load carrying capacity of swollen hydrated networks through matrix reinforcement [32–39]. The reinforcing phase is typically a stiff polymer (*e.g*., poly[ε-caprolactone] and poly[glycolic acid]) that is formed into a porous structure using techniques such as 3D printing [32], weaving [33], porogen leaching [40], or direct melt electrowriting [34–38].

There remain few attempts to explicitly design for poroelasticity and to our knowledge no demonstration of an engineered poroelastic material that approaches the physiological performance. In this work, we combine a hydrated network with a reinforcing phase; however, rather than simply transferring the compressive load to the stiffer reinforcing phase we use it to amplify IFP and demonstrate, to the best of our knowledge, the first engineered material system capable of exceeding isotropic linear poroelastic theory [12]. We refer to the composite as a fiber reinforced hydrated network (FiHy™). The primary objective of this study is to demonstrate the ability to produce IFP (flow-dependent) in an engineered material system and distinguish it from viscoelastic (flowindependent) effects. Our secondary objectives are to quantify the effects of fabrication strategy on the measured IFP and to develop a poroelastic model that informs the material design strategy.

## 2 Methods

Unless noted otherwise, all polymers and solvents were purchased from Sigma-Aldrich and Fisher Scientific respectively, and all cell culture supplies were manufactured by Gibco.

### 2.1 Scaffold fabrication

The scaffolds were composed of two phases: (1) fibrous polymer (modeling the role of the collagen fibers) and (2) hydrated network (modeling the role of the enmeshed proteoglycans). The fibrous phase was produced by electrospinning 12% (w/v) 80 kDa poly[ε-caprolactone] dissolved in a 3:1 (v:v) chloroform to methanol solution. The hydrated network was produced by electrospraying 10% (w/v) gelatin dissolved in 20% (v/v) acetic acid. Herein we refer to the fibrous phase as PCL and hydrated network as Gel.

**Fig. 1:**
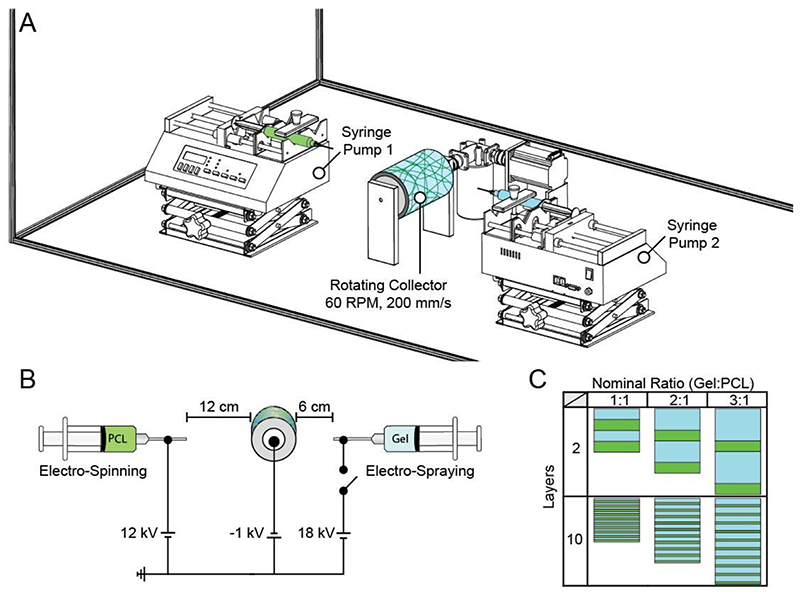
Fabrication of fiber reinforced hydrated networks via electrospinning and electrospraying. (A) The physical layout of the electrospinner. (B) Schematic of the combined electrospinning and electrospraying setup. Note that the 18 kV power supply switch is controlled by a 5 V signal from a programmable Arduino board and is toggled off during PCL deposition. (C) The primary variables of interest were the hydrated network content (Gel:PCL) and number of layers. As the Gel (blue) fraction increased, the total amount of PCL (green) remained constant. With increased layering the relative thickness of each layer became thinner to conserve the overall polymer volume. Note that the nominal ratio of Gel and PCL is shown here. The actual measured values are shown in Fig. 3A. (For interpretation of the references to colour in this figure legend, the reader is referred to the web version of this article.)

Producing a relatively thick mat with two different polymers and solvent systems required optimization of the polymer-solvent solutions, emitters and collector voltage, tip-to-target distances, and flow rates. In this study, the emitter voltages (kV), tip-to-target distances (cm), and flow rates (ml/h) were 12, 12, 2 for PCL and 18, 6, 2 for Gel (Fig. 1 and Table S7.1). These settings enabled scaffold fabrication under all Gel:PCL ratios (0:1 to 3:1) and constituent layering (2 to 100 layers) reported in this work. The collector was a 63.5 mm diameter aluminum drum covered by a single layer of aluminum foil, rotating at 60 rpm (~200 mm/s), and biased to -1 kV. The emitter was an 18-gauge blunt tip needle.

The amount of hydrated network and the number of layers were varied to determine their effect on IFP. The different nominal Gel:PCL ratios (v:v) were 0:1, 1:0, 1:1, 2:1, and 3:1. We fabricated scaffolds with *n* = 2, 5, 10, 50, or 100 layers. Note that *n* layers refer to *n* fibrous layers and *n* hydrated network layers. We fixed the total volume of PCL at 20 ml for all scaffolds except for the 1:0 ratio.

### 2.2 Microstructural analysis and composition

Scanning electron microscopy was performed on a Zeiss Auriga Cross Beam with an accelerating voltage of 5 kV. Samples were mounted to aluminum stubs using carbon tape and sputter coated in 15 nm of Cr. Images were taken of the exposed surface for each scaffold composition, see Fig. S7.1.

Nano-computed tomography (nanoCT) was performed on a single representative sample to quantify the structural anisotropy. The sample was hydrated in NiSO_4_ at 5% w/v in PBS for 2 h followed by air drying. Attempts to image hydrated networks were made using different aqueous buffers (deionized water, PBS, iodine solution, NiSO_4_ solution, and diluted ethanol); however, the attenuating effects of water were stronger than those of the FiHy™ networks. The sample was then placed in a 1.0 mm diameter Kapton® tube and scanned using a multiscale x-ray NanoCT Skyscan 2211. The 40 kV and 330 μA source produced a focal spot size of about 0.9 μm. Scans were acquired with an exposure time of 1300 ms, 2 frame averaging (no other filtering was applied), and 0.41°steps over a full 360°rotation. The final pixel size was 400 nm.

Images were reconstructed with NRecon v.1.7.4.6 software using the modified Feldkamp cone-beam reconstruction algorithm. A 394 ×180 ×200 μm volume of interest was selected from the center of the reconstructed image. A series of filtering, segmentation, and noise reduction steps were applied to isolate the fibers from the hydrated network. After post-processing, we quantified the 3D fiber orientation and pore space tortuosity (Fig. S7.11) in Avizo® 9.0.

Swelling of the FiHy™ networks was quantified by changes in mass. Dry samples were cut from the bulk using a 6 mm diameter biopsy punch and weighed on an analytical microbalance, 0.01 mg resolution. The samples were then placed under continuously flowing dry nitrogen for 24 h to further dry the samples. Following this, the samples were re-massed with an average 0.4% decrease in mass. Samples were then submerged in 0.25 ml of 1X phosphate buffered saline (PBS) and allowed to swell for 2 and 22 h under ambient conditions. The water wt% was calculated as:

(1) $$\text{Water}\;\text{wt}\;\text{\%}=\frac{\;\text{Hydrated}\;\text{Mass}\;-\;\text{Dry}\;\text{Mass}\;}{\;\text{Hydrated}\;\text{Mass}\;}\cdot 100\text{\%}$$

Due to the stochastic nature of electrospinning and electrospraying the true composition was assessed using a fluoraldehyde assay. Individual samples were cut from the bulk using a biopsy punch and weighed on an analytical microbalance. Samples were transferred to individual vials with 1 ml of PBS and heated to 60°C overnight to disrupt the networks. The solubilized sample and fluoraldehyde reagent were added to a 96-well plate in technical triplicate and read with an excitation/emission of 340/450 nm. In each plate a standard curve (0, 0.02, 0.1, 0.5, 1.0 mg/ml gelatin) was run in triplicate and used to quantify the gelatin wt%:

(2) $$\text{Gelatin}\;\text{wt}\;\text{\%}=\frac{\;\text{Gelatin}\;\text{Mass}\;}{\;\text{Dry}\;\text{Sample}\;\text{Mass}\;}\cdot 100\text{\%}$$

The wettability of dry FiHy™ scaffolds was determined by the contact angle using a dynamic surface analyzer. A 20 *μ*l drop of PBS was placed on the surface of a 6 mm diameter scaffold and imaged within 5 s. The contact angle was measured using the Low Bond Axisymmetric Drop Shape Analysis plugin [41] in ImageJ (version 1.47).

### 2.3 Reference samples

Bovine articular cartilage was used as a reference material for healthy articular cartilage. While human cartilage is a more relevant source, the poroelastic mechanics of bovine stifle cartilage have been shown to be similar to human knee cartilage and are a commonly used reference material [17,42]. Calf stifle joints were obtained from a local abattoir on the day of butchering. Osteochondral cores 16.5 mm in diameter were extracted from the trochlear groove and ridge. Specimens were washed in PBS and either used directly or frozen at -20°C. Frozen specimens were thawed under ambient conditions and submerged in fresh PBS for 2 h prior to cartilage isolation. Articular cartilage samples were isolated from the osteochondral cores using a biopsy punch and cut along the cartilage-bone interface. The cartilage cylinder was then placed in a V-block (see Fig. S7.12) to cut the bottom surface into a right cylinder. The articular cartilage surface remained intact.

Electrospun PCL and cast Gel were used as reference materials. Electrospun PCL was produced following the methods described in Section 2.1 *Scaffold Fabrication*. The electrosprayed Gel was not stable enough for mechanical testing. Instead, Gel was solubilized at 12% w/v in PBS at 60°C and cast in 35 mm diameter petri dishes.

### 2.4 Mechanical testing and analysis

The poroelastic response, poroelastic properties, repeatability, compression modulus, tension modulus, path length – distance fluid must flow to reach a free surface, and Poisson’s ratio were evaluated for FiHy™ scaffolds. Testing was performed on a TA Electroforce 3200 and 5500 equipped with uniaxial (tension-compression) load cells. Poisson’s ratio was evaluated on an Anton Paar MCR 302 rheometer.

Unless noted otherwise, samples were cut from the bulk material with a nominal diameter of 6 mm and hydrated in PBS for 2 h prior to testing. The thickness and diameter of hydrated samples were measured in triplicate with digital calipers (± 0.01 mm accuracy) before and after testing. All testing was performed under hydrated conditions, and a humidity chamber was used for all testing durations greater than 1800 s

#### 2.4.1 Poroelastic properties

Creep unconfined compression at 5 and 10 N (approximately 0.20 and 0.40 MPa) was used to quantify the poroelastic mechanics of the FiHy™ networks (*N* ≥ 3 for each composition and load). Samples were loaded at 0.5 V/s (volts per second) to the target load and held until equilibrium (10800 s) which we defined during preliminary testing as more than 10 times the creep time constant.

The voltage was used to directly control the crosshead as it gave a faster and more controlled response than displacement or force rate and maximized the transient poroelastic response. Samples achieved 90% of the target load within 1 s. Bovine articular cartilage was also loaded under these same conditions. Additional low-load creep compression tests at 0.04 (electrospun PCL) and 0.002 MPa (Gel) were conducted to avoid plastic deformation of the electrospun PCL and failure of the mechanically weaker gelatin.

The time-dependent relaxation for each sample was fit by a tension-compression poroelastic model for creep unconfined compression, see Section 2.5 *Poroelastic Modeling*. Additionally, a transversely isotropic poroelastic model for creep unconfined compression was used to validate and further describe the underlying physics, see Section 2.5 *Poroelastic Modeling*.

#### 2.4.2 Intra-sample repeatability

Creep unconfined compression at 5 N was used to evaluate intra-sample repeatability (*N* ≥ 1 per composition). Samples were loaded at 0.5 V/s and held for 10800 s followed by unloading for 10800 s. We then repeated the loading and unloading twice more and the fluid load fraction was calculated, see Section 2.5 *Poroelastic Modeling*.

#### 2.4.3 Compression modulus

The compression modulus (perpendicular to the fiber plane) was measured using ramp unconfined compression at 1, 5, 10, and 18 N (approximately 0.04, 0.20, 0.40, and 0.72 MPa). Samples (*N* ≥ 5 per composition) were loaded and unloaded at 0.5 V/s with 60 s of unloaded recovery following each loading-unloading. Each load condition was run in triplicate. The 18 N ramp was only run once as an attempt at specimen failure. Bovine articular cartilage was loaded under the same conditions to demonstrate the load carrying capacity of the native material. Additional load-sweeps at 0.5, 1, and 2.5 N were performed on the gelatin control. Each load was run in triplicate with the same loading rates and rest period.

Data were post-processed using a custom analysis script in MATLAB® 2018B. Contact was identified with a force threshold of 0.1 N. Force-displacement curves at 10 N and 1 N (Gel) were converted to stress and strain based on the initial cross-sectional area and thickness. A hyperelastic incompressible Neo-Hookean material model (Eq. (3)) was fit to the data to account for large strains,

(3) $$\sigma =\frac{E}{2(1+v)}\left(\lambda -\frac{1}{\lambda^{2}}\right)$$

where E is Young’s modulus of elasticity, *ν* is Poisson’s ratio, and *λ* is stretch.

#### 2.4.4 Tension modulus

Tensile testing of specimens (parallel to the fiber plane) was performed in duplicate using cam locking tensile grips. The setup and testing followed ISO 4952 (Methods of Test for Elastic Fabrics) as closely as reasonably possible. Rectangular samples were hydrated ~2 h prior to testing and measures of sample thickness and width were made in triplicate before and after testing. We loaded each sample (*N* ≥ 3 per composition) in the slack state and the grip-to-grip distance was measured; the true grip-to-grip distance was adjusted by the crosshead travel required to engage the specimen. The extension rate was 1.67 mm/s (this approximates the 500%strain/min as per the standard) to a target tensile stress of 0.3 MPa. The sample was then unloaded at 1.67 mm/s to 0.1 MPa. A total of 5 extension-retraction cycles were performed and then the sample was ramped to failure or the limit of crosshead travel. Based on preliminary measures, 0.1 to 0.3 MPa is approximately 25 to 75% of the yield stress. The extension-retraction cycling was designed to precondition the sample and eliminate any slip that might occur on the first few cycles. The tensile modulus was quantified by taking the last 3 cycles (extension and retraction) and performing a linear regression to the stress versus strain data.

#### 2.4.5 Path length

The fluid path length is modified to separate poroelastic (flow-dependent) from viscoelastic (flow-independent) effects. By altering the fluid path length, the time-dependent mechanics for a poroelastic material will change. The transient response of a viscoelastic material, which operates under molecular sliding and local rearrangement, is independent of path length [43,44]. The sample size or boundary conditions can be altered to take advantage of this distinguishing feature.

Unconfined compression was performed with porous and impermeable loading plates at 5 N (approximately 0.20 MPa). Samples (*N* = 2) were loaded at 0.5 V/s to the target load and held for 10800 s. Bovine articular cartilage was also loaded under these same conditions.

The porous loading plates were made of sintered bronze and were submerged in PBS prior to testing to reduce capillary forces from drawing fluid from the specimens. The permeability of the filter was measured using a direct permeation experiment with 7 replicates (Fig. S7.13). The permeability was found to be 4100 ± 500 mm^4^/(N · s).

#### 2.4.6 Poisson’s ratio

The undrained Poisson’s ratio was measured by compressing hydrated samples (*N* ≥ 2 per composition) to 0, 0.25, 1, and 5 N on a rheometer fitted with a transparent glass plate. Samples were first hydrated for 1 h in methylene blue to enhance contrast and then transferred to fresh PBS for an additional hr. Loads were applied using the initialization protocols provided in the rheometer software and typically took 10-30 s to achieve the target load. Once the target load was achieved an image of the deformed specimen was captured on an 8 MP camera. Images were post-processed using a custom MATLAB® 2018B script.

The radial strain (*ε_R_*) was calculated as:

(4) $$\varepsilon_{R}=\frac{d_{i}-d_{0}}{d_{0}}$$

where *d_i_* and *d_0_* are the deformed and undeformed diameter respectively. The undrained Poisson’s ratio was calculated by dividing *ε_R_* at a given load by the average normal strain (*ε_N_*) for *N* = 2 samples at the same load (Fig. S7.14).

### 2.5 Poroelastic modeling

The fluid load fraction (*F*’) is the typical metric for quantifying the poroelastic response of cartilage, and is defined as the fraction of the applied load supported by fluid pressure to the total applied load:

(5) $$F^{\prime }=\frac{F_{P}}{F_{P}+F_{S}}=\frac{F_{P}}{F_{T}}$$

where the summation of fluid load support (*F_P_*) and solid load support (*F_S_*) equals the total applied load (*F_T_*). Measuring fluid pressure or fluid load support directly is difficult; therefore, we use the effective compressive modulus (*E_C_*) and equilibrium compressive modulus (*E_s_*) to quantify *F*’ [45]:

(6) $$F^{\prime }=\frac{E_{C}-E_{S}}{E_{C}}$$

*E_s_* is the modulus once all fluid pressure has decayed and represents the support from the solid matrix and osmotic stress. *E_c_* is the modulus at any given time (Fig. S7.3) and is the summation of *E_s_* and fluid pressure. Therefore, taking the difference between *E_c_* and *E_s_* allows us to quantify the contribution of load support due to fluid pressure.

The fluid load fraction ranges from 0 to 1. A fluid load fraction of 0 represents the typical contact of ‘dry’ solids in which all the load is supported by solid stress. A value of 1 is the case of a hydrostatic bearing in which all the load is carried by fluid stress. In the following sections we briefly describe two poroelastic models developed in this work. Further details and their derivation are included in Supplementary Material.

#### 2.5.1 Tension-compression poroelastic model for creep unconfined compression

An analytical solution for creep unconfined compression of a poroelastic cylinder was developed based on the work of McCutchen [1] with an additional limiting elastic condition based on the work of Moore and Burris [46]. The model assumes phase incompressibility, material isotropy, small linear elastic strains, and impermeable and frictionless contact interfaces. These assumptions greatly simplify the model and allow us to arrive at a closed-form solution. The complete derivation of the model is provided in Supplementary Material.

The tension-compression (TC) poroelastic model was fit to creep unconfined compression data (deformation, time) using a nonlinear least-squares solver in MATLAB® 2018B.

#### 2.5.2 Transversely isotropic poroelastic model for creep unconfined compression

Additionally, we have developed a nonlinear (NL) poroelastic model that captures large deformations and variations in the permeability and porosity during compression. The model is based on that proposed by MacMinn et al. [47] but accounts for a transversely isotropic stress-strain relation following Cohen et al. [10]. The sample is assumed to remain cylindrical during compression due to frictionless contact with the platens, which substantially reduces the complexity of the model. Analytical expressions for the instantaneous and equilibrium response of the sample can be obtained. These are used to quickly extract the radial and axial Young’s moduli from experimental measurements of the mean axial strain by taking the Poisson’s ratios as known quantities. The permeability is then determined by minimizing the least-squares error between the model and data at specific values of the strain. An overview of the model, including the expressions for the instantaneous and equilibrium response, and the fitting procedure is provided in Supplementary Material; complete derivations will be the subject of future work.

### 2.6 Cytocompatibility

Cytocompatibility, based on metabolic activity, was performed on primary hMSCs [48]. FiHy™ networks were placed under vacuum for > 24 h to remove any residual solvents, weighed, and sterilized in 70% ethanol for > 18 h followed by three washes in sterile PBS over a period > 8 h. hMSC growth media was added to the sterilized FiHy™ networks at 5 ml/g of dry scaffold mass and incubated for 66 h at 37°C in a sealed falcon tube. Following incubation, the media was removed and stored in sterile vials at 4°C until testing.

Frozen hMSCs were passaged (passage number < 6), seeded in 96 well plates, and grown to ~80% confluency. hMSC media was prepared by adding 10% v/v hMSC-grade fetal bovine serum and 1% v/v penicillin-streptomycin to MEM alpha with GlutaMax. The media was removed and replaced with 100 μl of extracted media. At 24 and 72 h an alamarBlue assay was performed and quantified using a plate reader in fluorescence mode (560/590 nm). The plate included positive, untreated reference, and background control wells. Viability was quantified as:

(7) $$\text{Viability}\;(\text{\%})=\frac{\;\text{sample}\;-{\;\text{control}\;}^{+}}{\text{ref}\;-{\;\text{control}\;}^{+}}\cdot 100\text{\%}$$

Where *control*^+^ and *ref* are the positive control (cytotoxic) and untreated reference (100% viability) wells. The assay was performed with a minimum of 5 technical and 2 to 3 biological replicates. According to ISO 10993-5, the material shall be considered non-cytotoxic if the relative cell viability is ≥ 70%.

### 2.7 Statistics

Unless noted otherwise, all results are displayed as the mean ± 95% confidence interval. When possible, individual measures are shown to demonstrate variation in the data. Tests for significant differences were performed in Origin 2020. Significance was set at p < 0.05. The specific tests for significance are described throughout and in figure captions.

## 3 Results and discussion

### 3.1 Microstructural analysis and composition

**Fig. 2:**
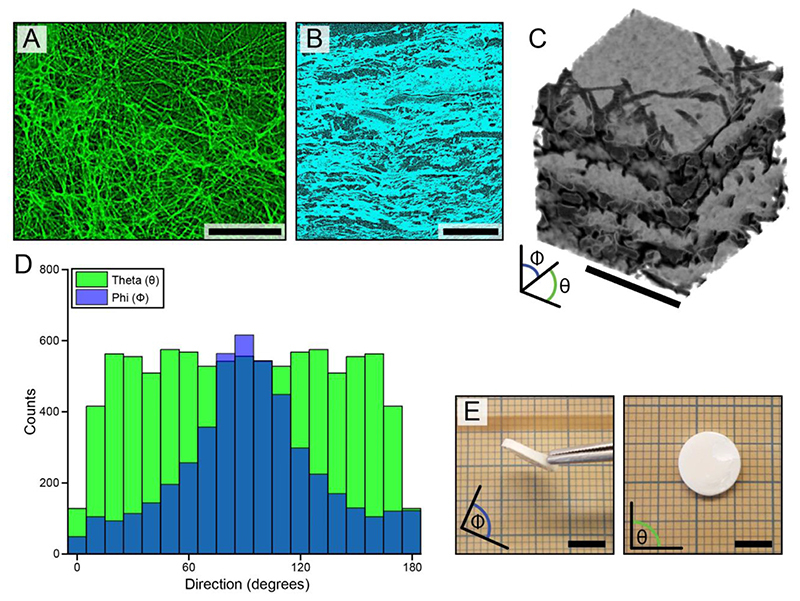
Fiber alignment for a representative FiHy™ scaffold (2:1 Gel:PCL ratio, 50 layers) using nanoCT. (A) Representative top-down and (B) cross-sectional (through thickness) views. Bright regions indicate signal attenuation due to polymer fibers. To aid in fiber visualization, 50 sequential images were summed in the top-down view and the maximum intensity projection of 34 sequential images was used for the transverse plane. (C) Magnified 3D reconstruction of the sample. (D) Spherical fiber alignment in the *θ* (tangent to the aluminum drum collector) and *Φ* direction. (E) Optical image of a representative FiHy™ scaffold (2:1 Gel:PCL ratio, 50 layers). Scale bars = 100 *μ*m (A and B), 50 *μ*m (C), and 5 mm (E).

The electrohydrodynamic process yielded a microstructure that consisted of 1.15 ± 0.55 *μ*m diameter PCL fibers (Fig. S7.1), 7.3 ± 1.8 *μ*m three-dimensional fiber-to-fiber distance, and a transversely isotropic fiber alignment (Fig. 2), with the transverse plane (*θ*) being tangent to the aluminum drum collector. Electrospinning of PCL was explicity chosen as it can produce fibers. Fibers offer nearly zero bending and compressive resistance unlike rods, beams, or plate-like microstructures. To demonstrate this, we assume that the compression of a pile of nonwoven fibers act as a network of simply supported beams with a central point load. The deflection or compliance for a simply supported beam is directly proportional to the third power of fiber length and inversely proportional to the fourth power of fiber diameter. Using the simply supported beam analysis, PCL fibers (Young’s modulus = 400 MPa) are expected to be ~8 times stiffer in bending than the collagen fibers found in articular cartilage (Young’s modulus = 1000 MPa; diameter = 0.05 *μ*m; fiber-to-fiber distance = 0.3 *μ*m [49]). However, typical 3D printed ‘fibers’ (Young’s modulus = 400 MPa; diameter = 100 *μ*m; fiber-to-fiber distance = 500 *μ*m) are ~1380 times stiffer in bending.

**Fig. 3:**
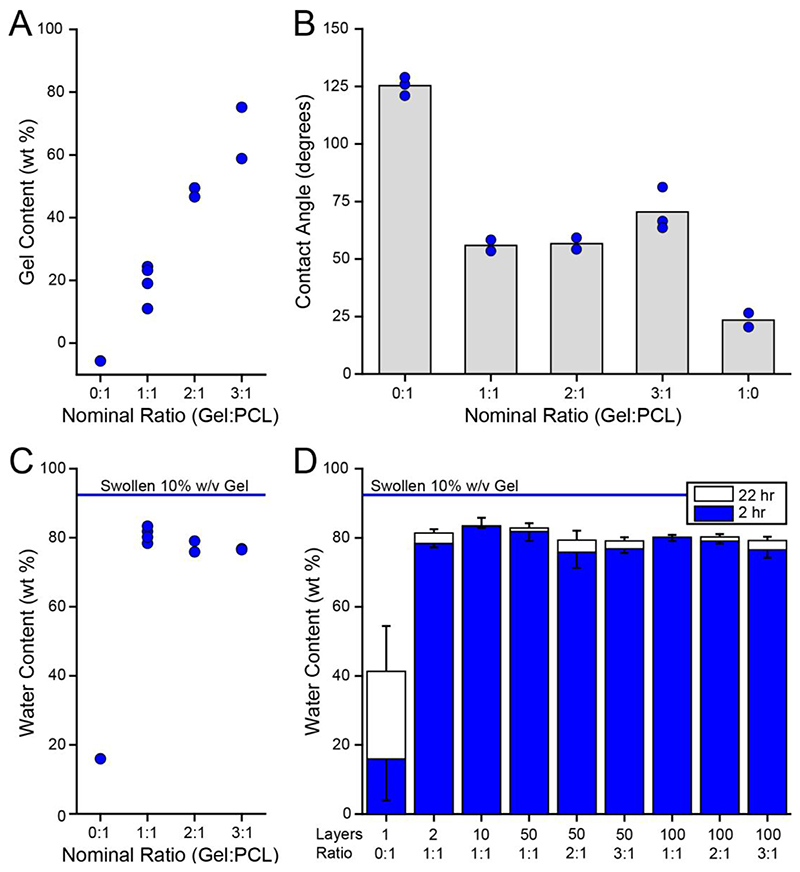
The composition and swelling capacity of FiHy™ scaffolds. (A) The measured Gel content for different Gel:PCL polymer ratios (*N = 2* to 4). (B) The dry contact angle (wettability) for different Gel:PCL polymer ratios (*N* = 2 to 3). Representative contact angle images are shown in Fig. S7.2. (C) The water content (swelling) for different Gel:PCL polymer ratios (*N* = 2 to 4). Note that *N* indicates the number of material replicates (different fabrication batches). Each *N* is composed of 3 technical replicates. (D) Effect of swelling duration for different scaffold compositions. For figure clarity, 2 h and 22 h of swelling are only shown with negative or positive error (95% confidence interval), respectively.

FiHy™ networks were characterized by their polymer content, wettability, swelling, and hydration time (Fig. 3). As expected, the measured Gel content for different scaffold compositions increased with the theoretical composition (Fig. 3A); however, variability existed within and across scaffolds of the same theoretical composition. We propose that this variability is caused by a non-uniform electric field during electrohydrodynamic deposition and results in biased polymer deposition. The hydrophilic and swelling capacity of the Gel increased wettability (Fig. 3B) and hydration of the FiHy™ networks (Fig. 3C). An additional 20 h of hydration only yielded an average 4.1% increase in water content relative to the 2 h timepoint (Fig. 3D); thus, all further experiments used a nominal 2 h hydration time.

### 3.2 Poroelastic mechanics

To evaluate poroelasticity, creep unconfined compression experiments were used to quantify the effective modulus over time (Fig. S7.3A). Following complete equilibration, the data were converted to fluid load fraction, Section 2.5 *Poroelastic Modeling*.

**Fig. 4:**
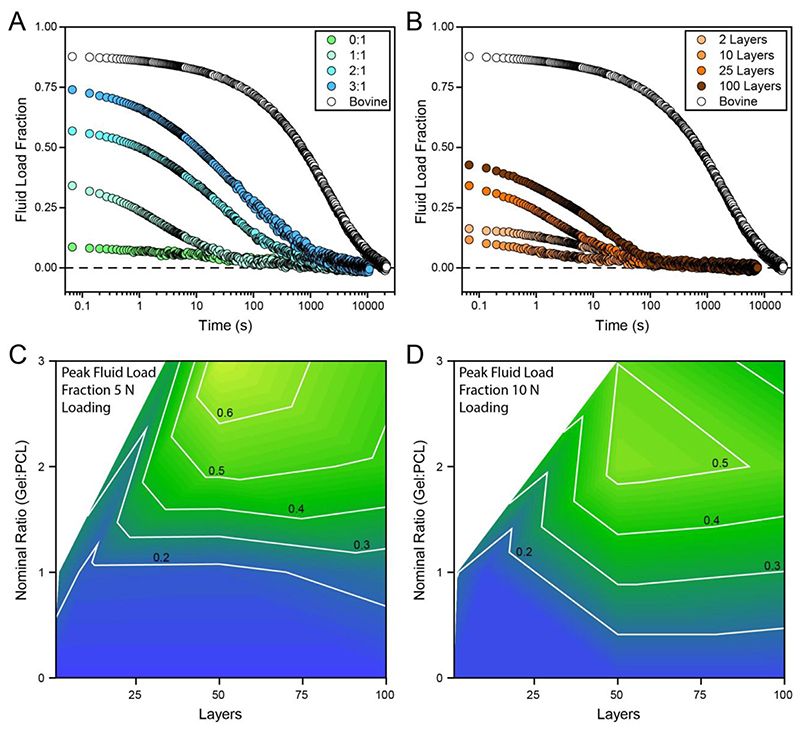
The poroelastic response for different FiHy™ compositions. (A) The Gel:PCL ratio is varied between 0:1 to 3:1 for 50 layers. (B) The number of layers ranges from 2 to 100 for a 1:1 Gel:PCL ratio. (A-B) A representative sample of bovine articular cartilage is tested under the same conditions to demonstrate the target response. (C) The peak fluid load fraction for 5 N and (D) 10 N unconfined creep compression. The peak fluid load fraction for 5 and 10 N loadings are also shown in Tables S7.2 and S7.3. Gel:PCL ratio significantly affected the peak fluid load fraction. The statistical tests and results are described in Table S7.2. *N* ≥ 3 for all compositions and loads.

As Fig. 4 demonstrates, FiHy™ networks are poroelastic and their degree of poroelasticity (fluid load fraction) can be controlled by the relative composition and layering approach. In general, it is observed that increased Gel:PCL ratios and greater layering are beneficial towards increasing the fluid load fraction. Peak values of 0.7 and 0.5 were reached under 5 N and 10 N creep loading respectively. The lower fluid load fraction at 10 N is hypothesized to be caused by yielding and damage to the microstructure; for this reason, we restrict any further analysis to the 5 N data.

Trends between fluid load fraction and the Gel:PCL ratio and layering suggest further exploration of the parameter space; however, there are practical limitations to this. Increasing the Gel:PCL ratio leads to a structure that begins to be dominated by the Gel phase and loses its load bearing ability, see Fig. S7.3. To maintain a mechanically relevant material, the Gel:PCL ratio was limited to 3:1. The layering effect was less apparent and more variable than constituent ratio but tended to suggest concurrent deposition would be an ideal fabrication strategy. It was not possible to use our configuration and settings as shown in Fig. 1 for concurrent deposition and future work will aim to address this.

Three consecutive creep tests were performed on each FiHy™composition to demonstrate intrasample repeatability. The peak fluid load fraction is shown in Fig. S7.4 and demonstrates a cycle-to-cycle variation on the same order as bovine cartilage. Interestingly, there was a trend of increasing fluid load fraction with each cycle. The cause of this observation is not known, but it may be linked to fiber reorganization, plastic deformation, or altered hydration between subsequent tests. Despite this, the sample maintains its ability to transfer load to the fluid phase over multiple cycles. Sample-to-sample variation is shown in Fig. S7.5 for a single FiHy™ composition. While peak fluid load fraction variations existed, the overall response was conserved across multiple samples (*N* = 5, 3:1 Gel:PCL ratio, 50 layers). The peak fluid load fraction, equilibrium compressive modulus, permeability, and modulus ratio were 0.68 ±0.04, 0.29 ±0.01 MPa, 0.13 ±0.06 mm^4^/(N·s), and 4.4 ±1.0, respectively. The material properties were derived from a poroelastic model fit described in Section 2.5 *Poroelastic Modeling*.

### 3.3 Ramp compression modulus

The ramp compression modulus of FiHy™ networks was quantified by fitting an incompressible Neo-Hookean material model to the data. The composite structure led to a modest increase in the ramp compression modulus that exceeded the sum of its parts (PCL mesh ~0.4 MPa and Gel ~0.004 MPa) with compressive moduli on the order of 1 MPa (Fig. S7.6). Layering was found not to have a significant effect on the compressive modulus, while the Gel:PCL ratio did demonstrate a significant effect. It should be noted that the 1:1 Gel:PCL ratio included 2, 10, 50, and 100 layer versions while 2:1 and 3:1 Gel:PCL ratios included 50 and 100 layer scaffolds.

### 3.4 Material properties from tension-compression poroelastic model

**Fig. 5:**
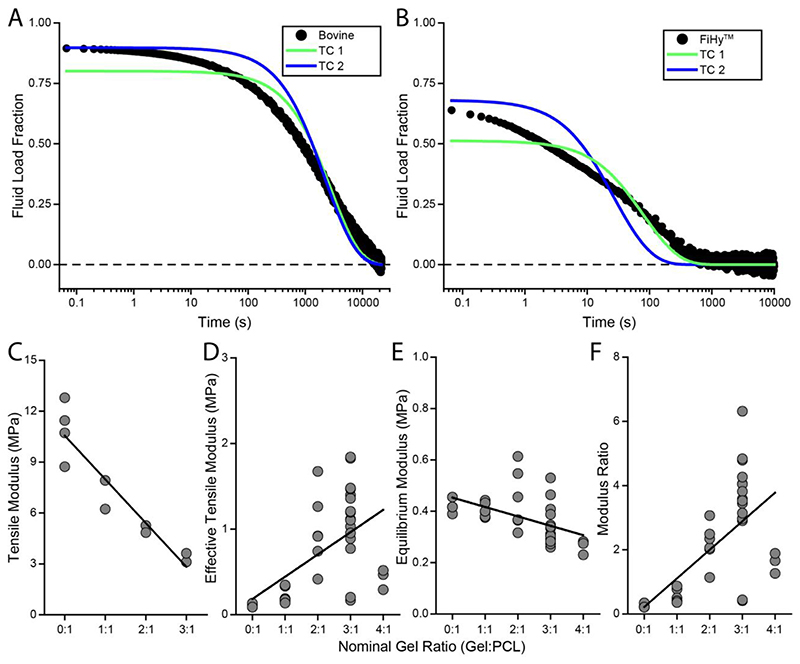
Aggregated material properties for 50- and 100-layer FiHy™ networks. The tension-compression poroelastic model is fit to (A) bovine articular cartilage and (B) a representative FiHy™ network. Two different fitting constraints (TC 1 and TC 2) are used to fit the model to the data. TC 1 leaves the tensile modulus and permeability unbounded while the equilibrium modulus is directly calculated from the equilibrated state. TC 2 leaves the permeability unbounded but forces the model to fit the first data point, which effectively sets the tensile modulus. While TC 2 produces a lower quality of fit (based on the residual sum of squares), it arguably provides a more accurate representation of the system by fitting the early time data, rather than biasing it toward equilibrium. TC 2 is used when discussing material properties derived from the model fit. (C) The tensile modulus is directly quantified from uni-axial tensile testing for different FiHy™ compositions. A linear regression (black line) indicates a significant reduction in modulus with increased Gel:PCL. *N* ≥ 2 specimens per composition. (D) The effective tensile modulus is quantified from the poroelastic model fits. A linear regression (black line) indicates a significant increase in tensile modulus with increased Gel:PCL. *N* ≥ 3 specimens per composition. (E) The equilibrium modulus is directly calculated as the modulus at the end of the creep test. A linear regression (black line) indicates a significant decrease in equilibrium modulus with increased Gel:PCL. *N* ≥ 3 specimens per composition. (F) The modulus ratio (tensile:equilibrium modulus) dictates the peak fluid load fraction and is derived from the poroelastic model fit. A linear regression (black line) indicates a significant increase in modulus ratio with Gel content. *N* ≥ 3 specimens per composition. Material properties derived from poroelastic fits for 0:1 and 1:1 Gel:PCL ratios should be interpreted cautiously as the model cannot provide a quality fit. A 4:1 Gel:PCL ratio (D-F) is included to demonstrate the effect of higher Gel content, and the consequential loss in material properties and poroelastic mechanics.

A tension-compression poroelastic model (TC), see Section 2.5.1 *Tension-Compression Poroelastic Model for Creep Unconfined Compression*, for creep unconfined compression was fit to the data to quantify the underlying material properties (effective tensile modulus, equilibrium modulus, modulus ratio, and permeability). Fig. 5A and 5B demonstrate two different fitting constraints (TC 1 and TC 2) applied to bovine articular cartilage and a representative FiHy™ scaffold. TC 2 is used throughout the manuscript as it forces the model to fit the early time data and is more representative of the overall response.

Key material properties are shown in Fig. 5C–5F. Fig. 5C is the experimentally derrived tensile modulus from uniaxial tensile testing, demonstrating that incorporating a weaker phase (Gel) decreases the tensile modulus of the structure. Interestingly, the effective tensile modulus which is derived from the TC model (Fig. 5D) directly opposes the uniaxial tensile modulus. In addition, the tensile modulus between the two methods is separated by about an order of magnitude. This seemingly contradictory result appears to be demonstrating an important design principle.

Recalling that the Gel is the swellable network, it is responsible for volumetric changes in the sample. This swelling stress is balanced by the fiber stress, which induces a pre-stress that straightens the fibers and reduces the deformation required to engage the fibrous network and build fluid pressure [50]. Therefore, we hypothesize that while an increase in the Gel:PCL ratio reduces the uniaxial tensile modulus, it provides a greater fiber pre-stress, and thus a greater capacity for IFP. The modulus ratio (effective tensile:equilibrium compressive modulus) determines the peak IFP (Fig. 5F) and is found to increase with the Gel:PCL ratio.

Interestingly, the permeability (Fig. S7.7) did not significantly decrease with greater Gel fractions. In fact, it has a slightly increasing trend with greater gel fractions. Two key factors may contribute to this result. First, the layered nature of the composite may provide regions of Gel that are not embedded and constrained by a fibrous network and may yield a larger mesh size. As the layers become thicker this effect could become more pronounced. Second, the model is formulated on the mechanism of poroelasticity and thus performs poorly when attempting to fit material responses that are not dominated by poroelasticity. Specifically, viscoelasticity may contribute to the response, and even dominate the response for non-poroelastic variations (*e.g*., 0:1). Since viscoelasticity is not considered in the model, the permeability term will capture its temporal effect. Regardless, the results demonstrate a limit to the Gel:PCL ratio and suggest an alternative hydrated network may be required to reach physiological values.

### 3.5 Path length

**Fig. 6:**
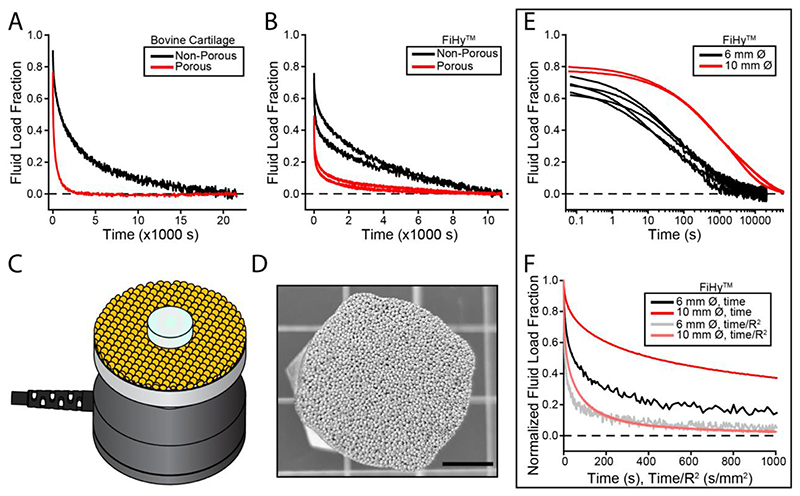
Path length experiments involving impermeable (non-porous) and permeable (porous) interfaces were conducted in unconfined compression. (A) The effect of path length for bovine cartilage (*N* = 1) and (B) FiHy™ scaffolds (2:1 Gel:PCL, *N* = 2). The non-porous platen was formed from stainless steel while the porous platen was formed from sintered bronze. (C) Schematic of the porous platen used for testing path length effects. (D) Image of sintered bronze platen. Scale bar = 5 mm. (E) The path length was further investigated using two different sample diameters 6 and 10 mm (nominal) in unconfined compression. This approach eliminates effects from differences in interface topography and dissimilar materials (stainless steel versus bronze). *N* = 2 samples at 10 mm and *N* = 5 samples at 6 mm diameter are shown to demonstrate repeatability. (F) The fluid load fraction from (E) was normalized and the time axis was truncated to visualize the transient response. One representative 10 mm and 6 mm diameter sample from (E) are shown. Dividing time by the square of the characteristic path length (radius, R) collapsed the data on to a single curve, which is the expected result from poroelastic theory.

Until now, we have assumed that the time-dependent changes are of poroelastic origin rather than viscoelastic. However, it is critically important to identify which mechanism is active in FiHy™. One approach to decouple poroelasticity from viscoelasticity is to alter the path length to the free surface. A shorter path length will allow poroelastic materials to equilibrate more quickly. In Fig. 6A and 6B porous compression platens 5 to 6 orders of magnitude more permeable than the material of interest were compared to impermeable platens. For both articular cartilage and FiHy™ samples, the porous platens led to faster equilibration rates compared to the non-porous conditions. In a follow up experiment using impermeable platens, the nominal sample diameter was reduced (from 10 to 6 mm) to alter the radial path length for fluid flow. As Fig. 6E demonstrates the smaller sample diameter equilibrates more quickly which agrees with poroelastic theory. According to poroelastic theory, the time constant is proportional to the square of the characteristic dimension (sample radius) [51,52]. Therefore, as Fig. 6F shows, dividing time by the square of the characteristic dimension collapses the data onto a single curve further demonstrating consistency with poroelastic theory. The material properties for both sample dimensions are quantified in Table S7.4.

### 3.6 Poisson’s ratio

Measures of Poisson’s ratio for FiHy™ were found to be unreliable below normal strains of ~0.25 mm/mm (Fig. S7.8). At normal strains greater than 0.25 mm/mm the mean Poisson’s ratio approached zero (0.0 ±0.06, mean ±95% confidence interval), which is in agreement with the superficial zone of cartilage (*ν* = 0.035) [53]. The quantitative agreement with the superficial zone likely stems from the tangential fiber alignment in both materials [54]. Given that it took nearly 30 s of loading to reach each target condition the reported values represent a semi-drained Poisson’s ratio rather than an instantaneous or drained condition [55]. We hypothesize that at equilibrium the value will be no greater than the semi-drained condition and thus report a Poisson’s ratio of 0.

### 3.7 Constitutive modeling

**Fig. 7:**
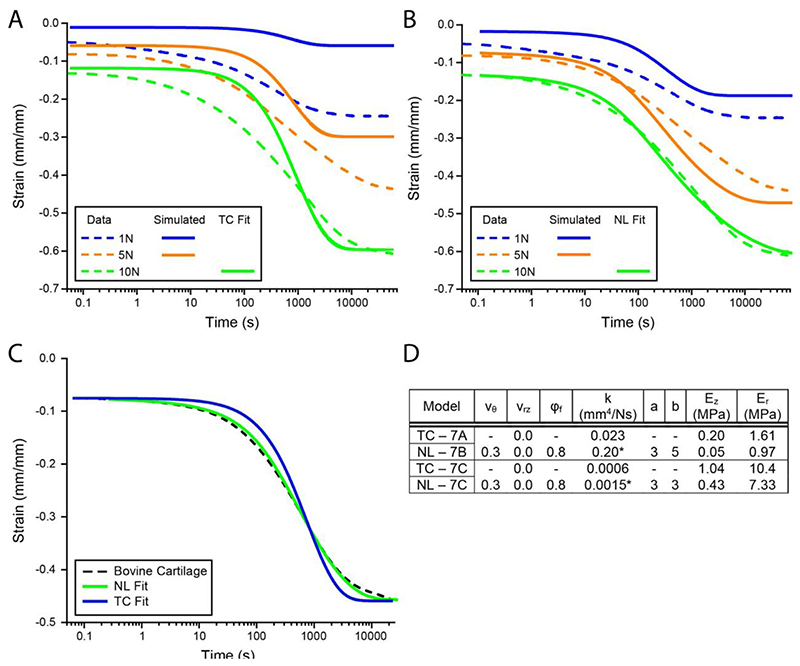
Three creep loads were applied to the same FiHy™ sample (3:1 Gel:PCL). The (A) TC and (B) NL models were fit to the 10 N experimental data. The resulting material properties were then used to predict the creep response for 5.0 and 1.0 N. Experimental data are shown as (dotted lines). Simulated data for 1.0 and 5.0 N unconfined compression are shown as solid lines. (C) The NL and TC models were fit to bovine articular cartilage. (D) Material properties from the TC and NL model fits. Note the additional terms: *v_θ_* (angular Poisson’s ratio), *v_rz_*, (rz Poisson’s ratio), and *φ_f_* (fluid fraction). *For the NL model the permeability value is given for the unstressed reference condition *k_0_* and nonlinear coefficients *a* and *b*. (-) indicates that a material property is irrelevant to the model. The material properties are also listed in Tables S7.5 and S7.6.

Our goal in developing a poroelastic model was to guide the design, quantify material properties, and predict the material response. The TC model provided sufficient design criteria, ability to fit the data, and extract material properties. In Fig. 7A the TC model was fit to the 10 N loading condition. These material properties were then used to predict the 5.0 and 1.0 N responses. Unfortunately, the predictive power of the TC model was insufficient. While there are several potential reasons for this discrepancy, we focused on developing a more accurate physical description of the system. A nonlinear transversely isotropic poroelastic (NL) model with strain-dependent permeability was derived (see Section 2.5 *Poroelastic Modeling*) and fit to the same data in Fig. 7B. The predictability of the NL model was substantially greater when compared to the TC model. We propose the NL model was a better physical descriptor as it accounted for the large strains (nonlinear elasticity), transversely isotropic fiber orientation, and consolidation (strain-dependent permeability).

The NL and TC models were then fit to a single data set for creep unconfined compression of bovine cartilage, see Fig. 7C. The fits demonstrate that both models are capable of explaining the data. The NL fit appears superior given its lower residual sum of squares. Comparing the material properties from both models we can observe several important results. The TC model gives a greater value for the equilibrium compressive modulus (defined as E_z_ for the NL model). This is expected as Hencky (or logarithmic) strains are used in the NL model. The reported equilibrium compressive modulus of bovine articular cartilage is 0.6 ±0.2 MPa [56] which is in general agreement with the TC (1.0 MPa) and NL (0.4 MPa) models.

The effective tensile modulus (defined as E_r_ for the transversely isotropic model) is greater than E_z_ by approximately an order of magnitude for both models and materials shown in Fig. 7. This tension-compression nonlinearity or transversely isotropic structure enables the relatively compliant FiHy™ and cartilage to achieve high levels of IFP, fluid load support (Fig. 4), and a greater effective modulus (Fig. S7.6). Bovine articular cartilage has been shown to have an effective tensile modulus of 13 ± 2 MPa [56] which is in good agreement with the TC (10 MPa) and NL (7 MPa) models.

The permeability differs between the two models due to the inclusion of strain-dependent permeability in the NL model. The value reported from the NL model is the unstrained permeability value, while the TC model which assumes constant permeability must fit to the overall response and likely approximates the true permeability around 50% deformation. We can attempt to equate these permeability values by taking 50% of the strain magnitude in Fig. 7C and assume that the axial strain is only associated with fluid exudation. Therefore, at time = 0 we have 80% fluid and at the intermediate strain we have 62.5% fluid. Applying this along with the unstrained permeability value (0.0015 mm4/(N · s)) and scaling coefficients (a = 3 and b = 3) we calculate a permeability value of 0.0007 mm4/(N · s) which is in excellent agreement with the TC model (0.0006 mm^4^/(N·s)) and work of others (0.0006 mm^4^/(N·s)) [56].

Both models demonstrate an ability to fit the experimental data, and the resulting material properties align with the existing literature. The choice of model will depend on the application and needs of the user. While the TC model offers rapid fitting and the ability to capture the first-order deformation response of cartilage and FiHy™, the NL model is superior in considering nonlinear elasticity and strain-dependent permeability, which enables greater predictability. Extending the TC model to nonlinear elasticity and strain-dependent permeability will be the focus of future work.

### 3.8 Cytocompatibility

**Fig. 8:**
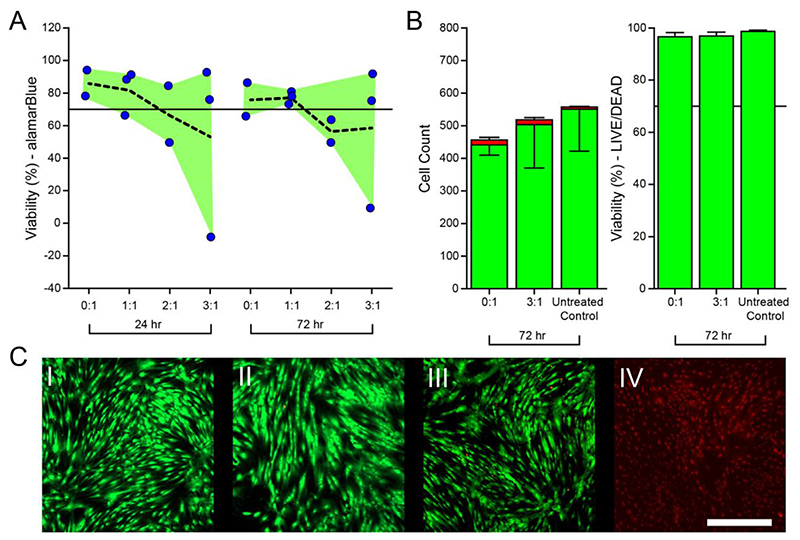
(A) Cytocompatibility with hMSCs was quantified using the alamarBlue metabolic assay. Extracted media was placed in direct contact with cells for 24 or 72 h of static culture. Dashed line indicates the mean response. *N* = 2 to 3 biological replicates are included for each condition. According to ISO 10993-5, the material shall be considered non-cytotoxic if the relative cell viability is ≥ 70% (horizontal line). (B) LIVE/DEAD™ quantification of hMSCs at 72 h, mean ±standard deviation. Live and dead cell numbers were counted manually for each image (1 image per well, 3 images quantified per condition). Viability was quantified based on the number of live cells to the total cell count. (C) Representative LIVE/DEAD™ images for (I) 0:1, (II) 3:1, (III) untreated control, (IV) and positive control conditions after 72 h. The signal intensity for IV was increased for visualization purposes. Scale bar = 500 *μ*m.

While FiHy™ networks were composed of biocompatible polymers, the solvents used during the fabrication process were not. In principle the solvents should come off during the electrospinning and electrospraying process maintaining scaffold cytocompatibility. To verify cytocompatibility, soluble compounds were extracted and added to ~80% confluent primary human mesenchymal stem cells (hMSCs) for 24 and 72 h. Fig. 8 shows the cytotoxicity results, with cytotoxic defined as a mean viability of less than 70%. Surprisingly, 2:1 and 3:1 Gel:PCL scaffolds fell within the cytotoxic range.

To confirm this was a cytotoxic effect, a LIVE/DEAD™ assay was performed on a subset of the wells and indicated low dead cell counts (Fig. 8B). The percentage of dead cells were 1 ± 1%, 3 ± 2%, and 3 ± 1% for the untreated control, neat PCL (0:1), and the highest Gel concentration (3:1). The results suggest that rather than being directly cytotoxic there was a reduction in metabolism which correlated with increased Gel content.

To determine the role of Gel content on viability, gelatin was solubilized in hMSC media at 0.1 g/ml to 0.00005 g/ml at 37°C. alamarBlue™ and LIVE/DEAD™ viability testing were performed at 24 and 72 h. The results (Fig. S7.9) demonstrate a dose-dependent response on viability when measured via alamarBlue™; however, this is not observed in the LIVE/DEAD™ assay (Fig. S7.10). This further supports our hypothesis that solubilized Gel is limiting metabolic activity rather than killing the cells. While this generally demonstrates cytocompatibility, the dissolution of the Gel phase at physiological conditions will degrade the poroelastic mechanics and render the design ineffective. Addressing this shortcoming will be critical in future work that aims to design a physiological implant. Potential approaches with the existing formulation include the use of a carbodiimide coupling solution (*e.g*.,EDC), a primary amine crosslinker (*e.g*., formaldehyde), or prefunctionalization with a reactive group (*e.g*., methacryloyl).

In this work we unambiguously demonstrate poroelasticity in an engineered composite by distinguishing the time-dependent response from viscoelasticity. While FiHy™ does begin to approach physiological values for fluid load support, it is limited by its effective compressive modulus (1 MPa) and rapid fluid exudation (0.1 mm^4^/(N·s)). Increasing the stiffness of the composite requires greater load transfer to the fluid phase. To achieve this the poroelastic models tell us that the effective tensile modulus needs to increase. This is also supported by existing experimental and theoretical literature investigating the role of the collagen [57] or fiber network [58,59]. Amplifying the effective tensile modulus is achievable by using a higher fiber fraction or a greater fiber modulus. In this work we span a fiber content from 100% (0:1 Gel:PCL) to 0% (1:0 Gel:PCL) and found an optimum at 3:1 Gel:PCL. Therefore, future work will target increased fiber modulus using a stiffer polymer such as poly(lactic acid). An alternative or additional approach could include bonding this material to a rigid substrate, which produces lateral confinement and amplifies poroelastic fluid pressures [58]. In addition to modifying the effective tensile modulus, a lower permeability matrix is necessary to reduce the rate of fluid exudation, and thus prolong IFP. This can be achieved by developing a smaller effective pore size, increasing the density of the matrix, and using a higher molecular weight water-swollen polymer [60,61]. Finally, cartilage is known for its depth-dependent fiber alignment and distribution of constituents which have been shown to contribute to a host of mechanical parameters [62], ultimately mimicking the full functional response of articular cartilage will likely require the consideration of these additional features.

## 4 Conclusion

In this work we have developed the first engineered material that exceeds isotropic linear poroelastic theory, demonstrated control over the poroelastic performance, and begins to approach physiological poroelasticity. The system was composed of fibrous electrospun poly(*ε*-caprolactone) and enmeshed electro-sprayed gelatin. Both the constituent ratio and layering were significant drivers of poroelasticity. Furthermore, we decoupled poroelastic from viscoelastic characteristics. The constituents chosen for this work were intended to demonstrate an experimental proof of the extensive theoretical literature on poroelastic mechanics. More importantly, this work provides a logical foundation, rational design approach, and relevant characterization scheme for developing cartilage mimics that generate IFP. We hope that this work will serve as a blueprint for future design approaches of cartilage mimics that produce IFP.

## Supplementary Material

Supplementary material

## Statement of significance

Poroelasticity drives the functional mechanics of articular cartilage (load bearing and lubrication). In this work we develop the design rationale and approach to produce a poroelastic material, known as a fiber reinforced hydrated network (FiHy™), that begins to approach the native performance of articular cartilage. This is the first engineered material system capable of exceeding isotropic linear poroelastic theory. The framework developed here enables fundamental studies of poroelasticity and the development of translational materials for cartilage repair.
