# Supplementary material for: Fiber reinforced hydrated networks recapitulate the poroelastic mechanics of articular cartilage
Source: Acta Biomater. Author manuscript; Available in PMC 2024 Dec 9. (PMC7617126; doi:10.1016/j.actbio.2023.06.015)
Supplement: Supplementary material [file EMS193193-supplement-Supplementary_material.docx]

Fiber Reinforced Hydrated Networks Recapitulate the Poroelastic Mechanics of Articular Cartilage

A.C. Moore^1^

M.G. Hennessy^2,3^

L.P. Nogueira^4^

S.J. Franks^5^

M. Taffetani^2,3^

H. Seong^1^

Y.K. Kang^1^

W.S. Tan^1^

G. Miklosic^1^

R. El Laham^1^

K. Zhou^1^

L. Zharova^1^

J.R. King^5^

B. Wagner^6^

H.J. Haugen^4^

A. Münch^2^

M.M. Stevens^1*^

Author Affiliation:

^1^Department of Materials, Department of Bioengineering and Institute of Biomedical Engineering, Imperial College London, London, UK

^2^Mathematical Institute, University of Oxford, OX2 6GG, Oxford, UK

^3^Department of Engineering Mathematics, University of Bristol, BS8 1TW, Bristol, UK

^4^Department of Biomaterials and Institute of Clinical Dentistry, University of Oslo, Oslo, NO

^5^School of Mathematical Sciences, University of Nottingham, NG7 2RD, Nottingham, UK

^6^Weierstrass Institute for Applied Analysis and Stochastics, D-10117, Berlin, DE

7.0 Supplementary Material


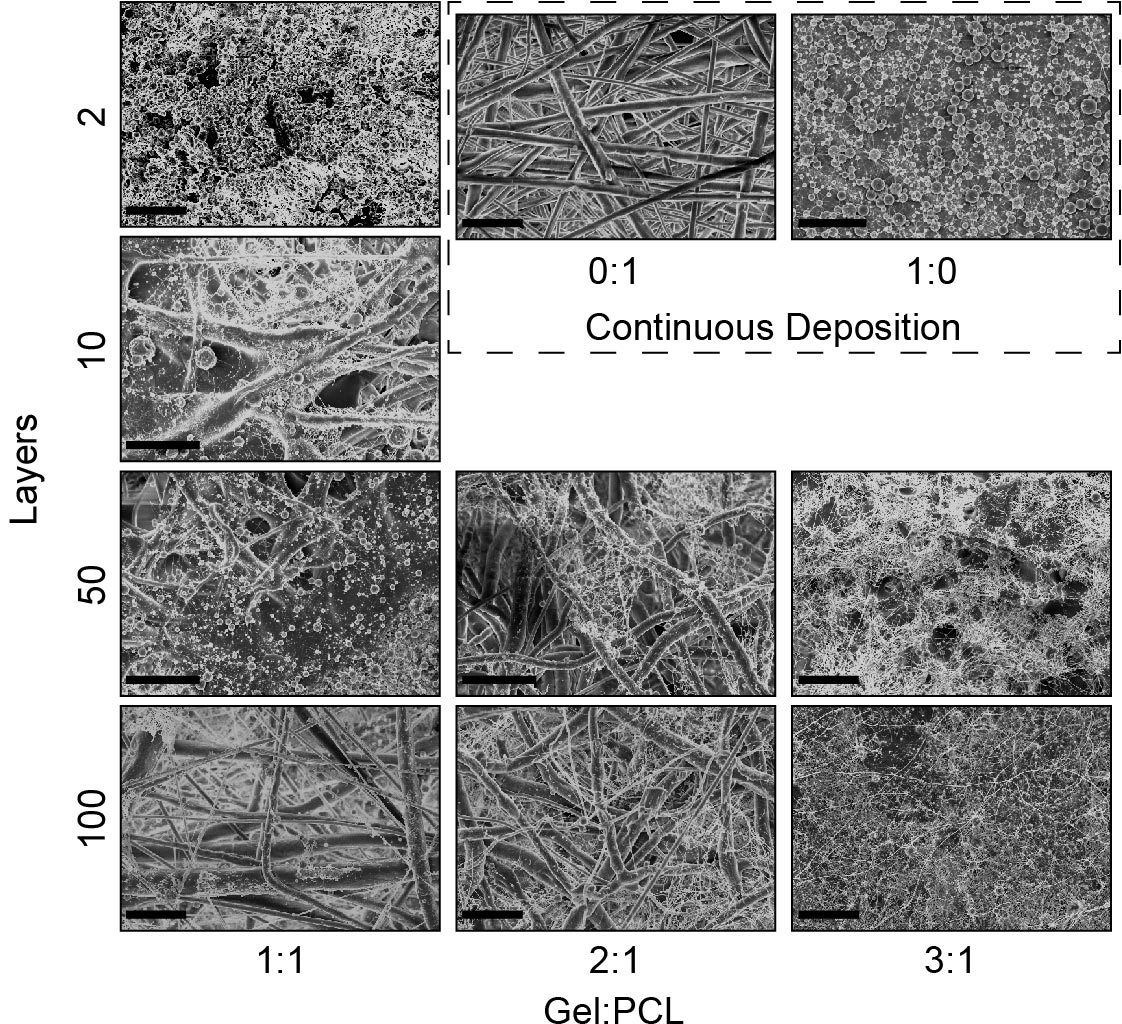


**Figure S7.1** Scanning electron micrographs of representative regions for each fabrication condition tested. Single constituent electrospun PCL (0:1) and electrosprayed Gel (1:0) are shown in the dashed box and were continuously deposited. PCL fiber diameter was measured manually in ImageJ. 221 individual measures were made of PCL fibers resulting in a mean and median fiber diameter of 1.15 and 1.14 μm. Gel particles were measured in ImageJ using the Analyze Particle function. The mean and median particle diameter were calculated as 0.34 and 0.17 μm. Scale bars = 10 μm. Gel was the last layer to be deposited and is thus the most visible for few layers and high Gel:PCL ratios.


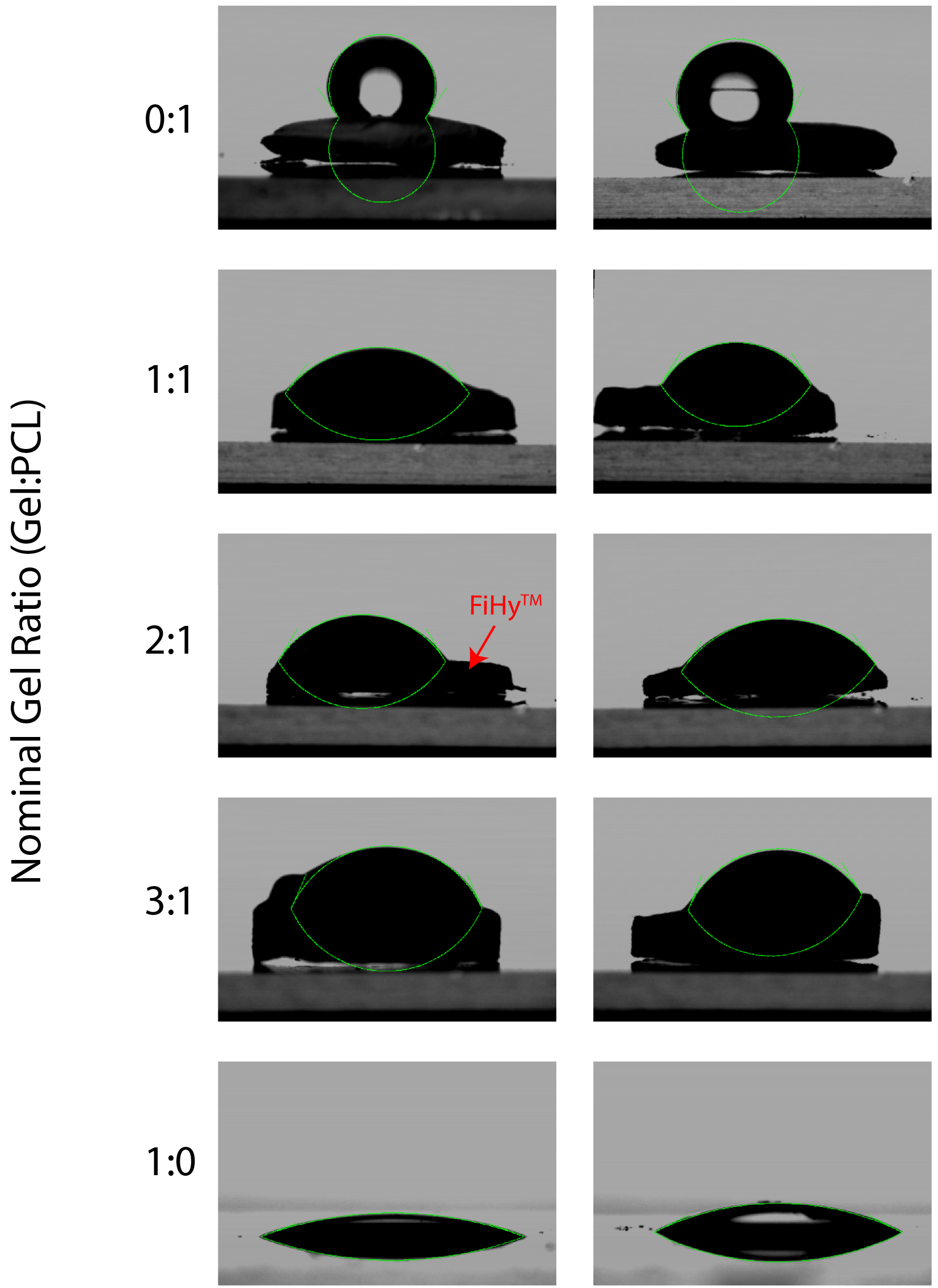


**Figure S7.2** Contact angle images and fits (green outline) for different FiHy™ networks. A 20 µl drop of PBS is placed on FiHy™ samples. Two representative images are shown for each composition. The width of each image is approximately 8 mm.


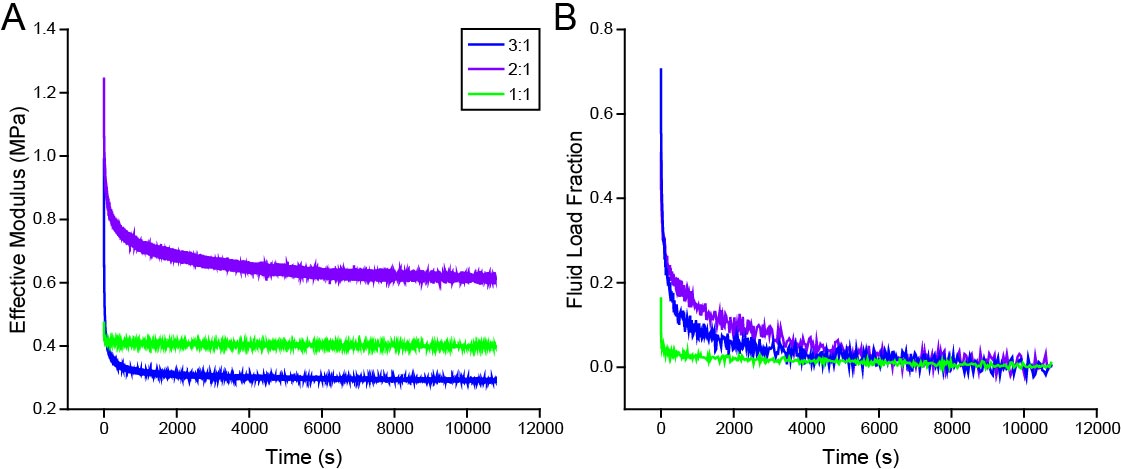


**Figure S7.3** Effective compressive modulus and fluid load fraction versus time. Note that a 2:1 Gel:PCL ratio is stiffer; however, the 3:1 Gel:PCL ratio produces a greater peak fluid load fraction. This loss in load carrying capacity limits the benefit of higher Gel:PCL ratios.


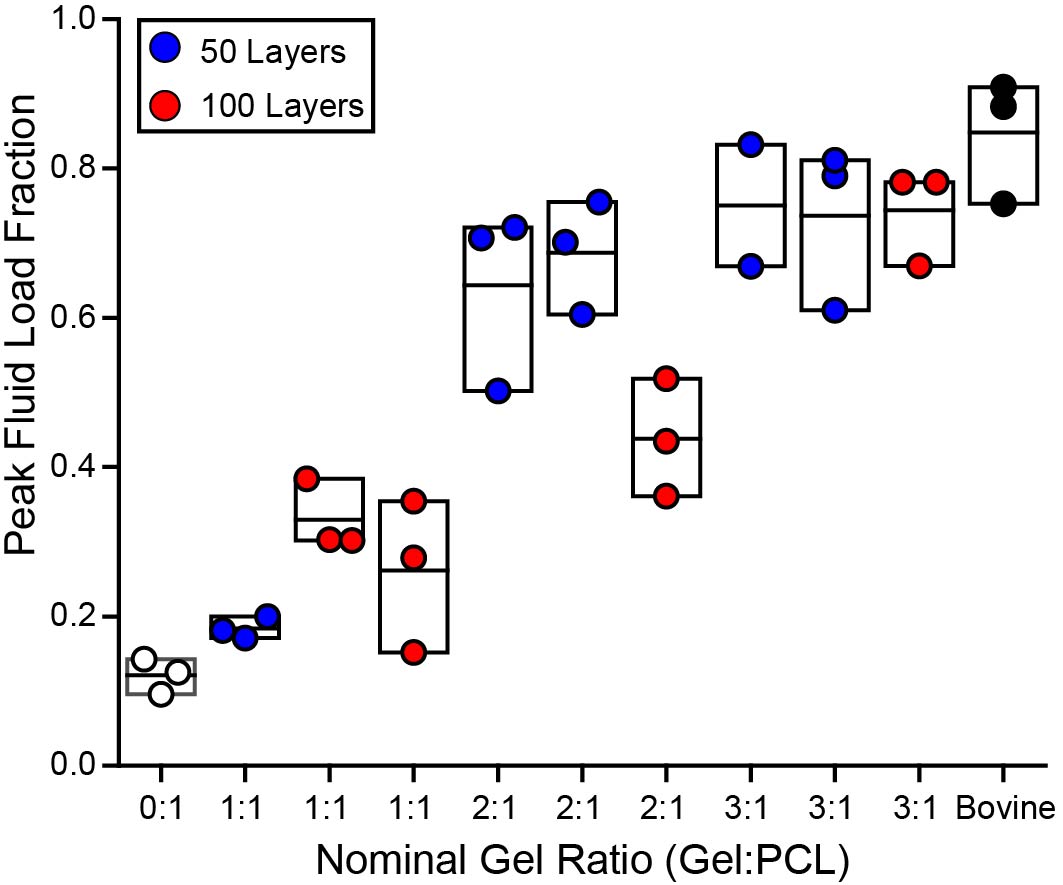


**Figure S7.4** Intrasample peak fluid load fraction repeatability for different Gel:PCL ratios and layers. The mean (line) and range (box) are shown. Three repeats are shown for each test. Note that some compositions and layers were tested more than once to demonstrate sample to sample repeatability.

**
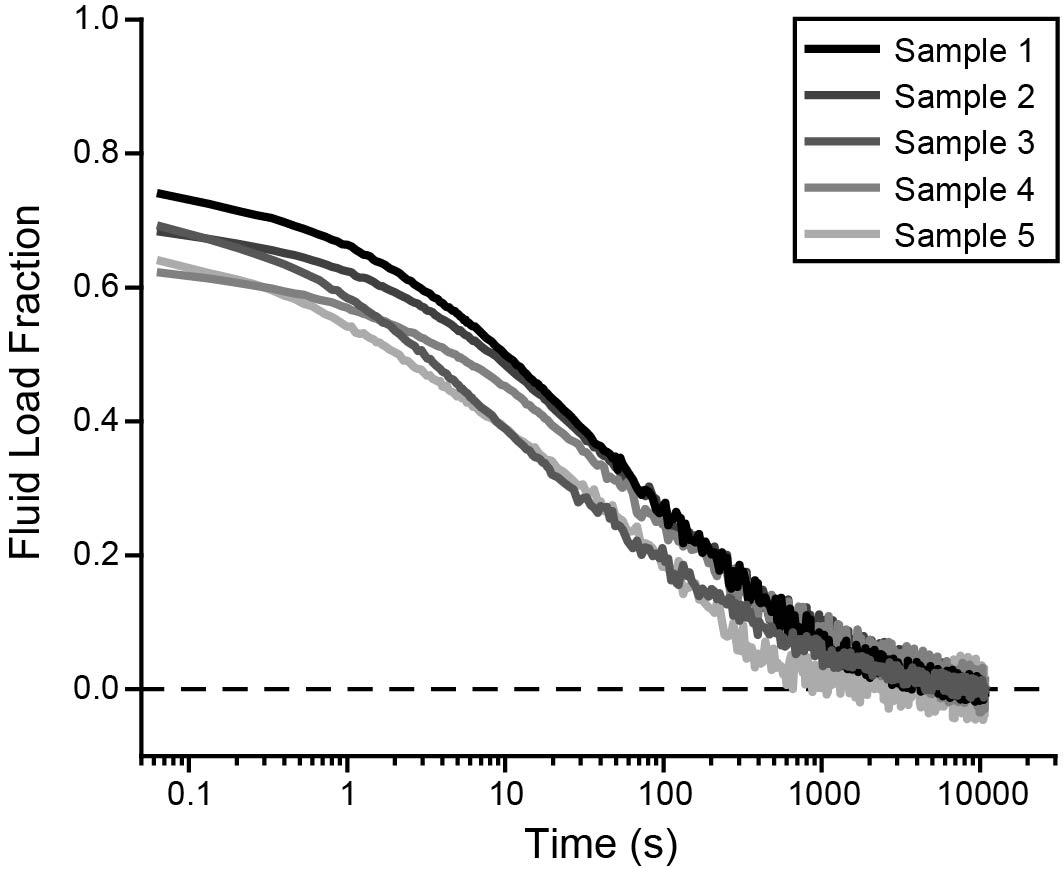
**

**Figure S7.5** Intersample repeatability for *N* = 5 samples (3:1 Gel:PCL ratio, 50 layers).


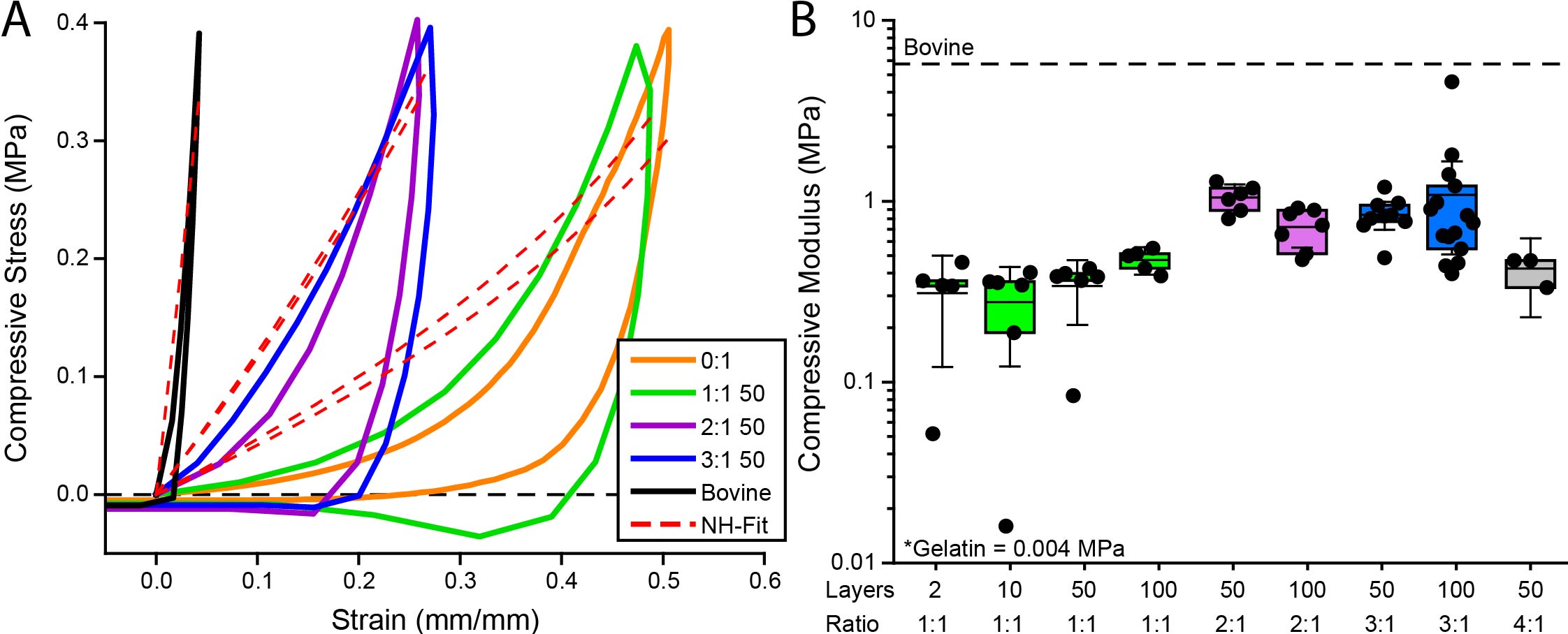


**Figure S7.6** (A) Representative ramp unconfined compression for different FiHy™ scaffolds. The Neo-Hookean hyperelastic model is fit to the approach. The model fits poorly to the 0:1 and 1:1 FiHy™ scaffolds and thus compressive modulus values should interpreted with caution. (B) The compressive Young’s modulus was determined by the Neo-Hookean model fit. The mean (line), 25^th^ to 75^th^ percentile (box), and 95% confidence interval (error bars) are shown with independent samples (dots), *N* ≥ 3. A Two-Way ANOVA detected significant effects from the Gel:PCL ratio (p < 0.034) but not number of layers (p < 0.95).


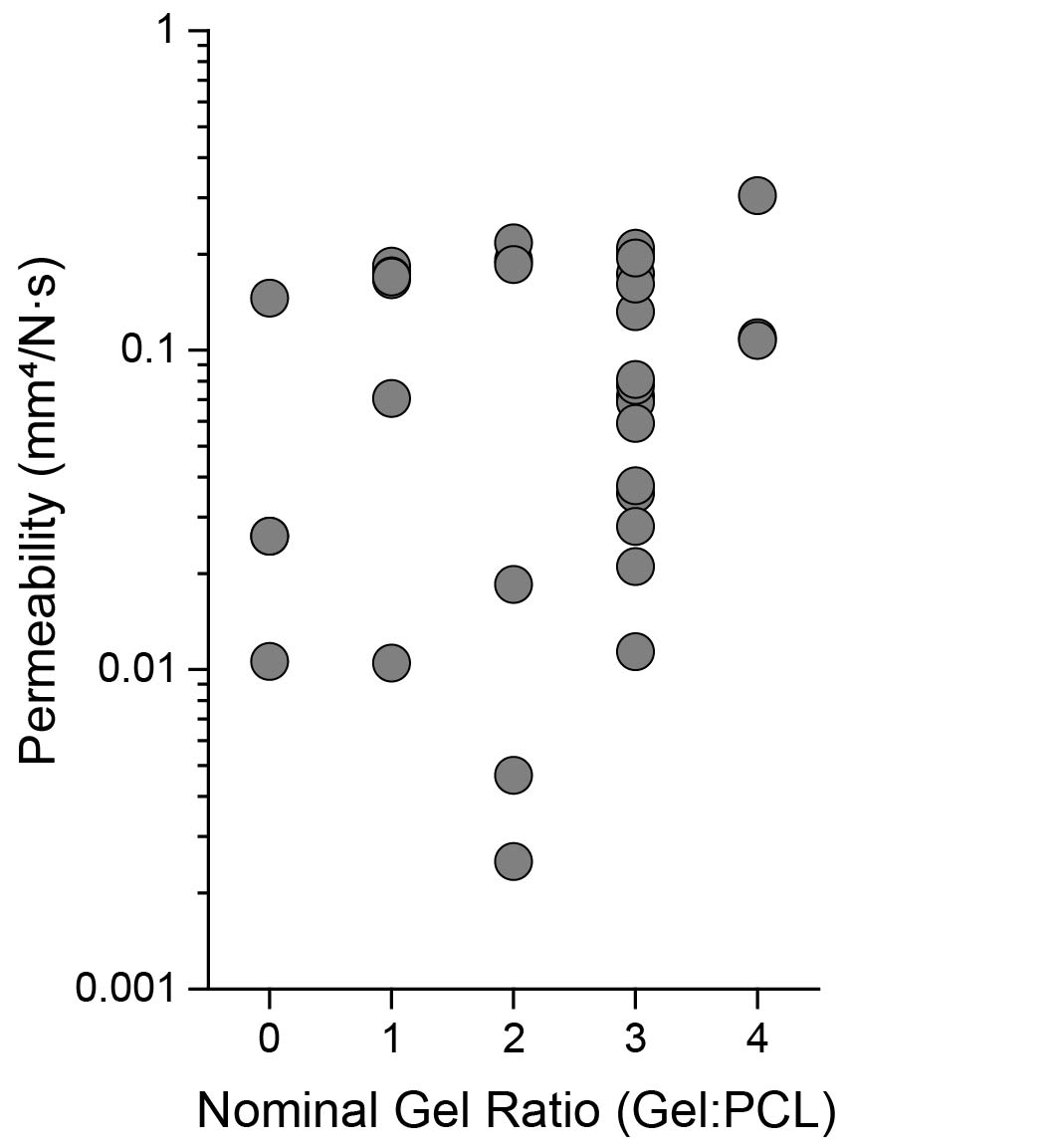


**Figure S7.7** The permeability is quantified from the TC model fit. There was a trending but nonsignificant increase in permeability with increased Gel:PCL. *N* ≥ 3 specimens per composition. Permeability values at 0:1 and 1:1 Gel:PCL ratios should be interpreted with caution as the model cannot provide a quality fit. A 4:1 Gel:PCL ratio is included to demonstrate the effect of higher Gel content.


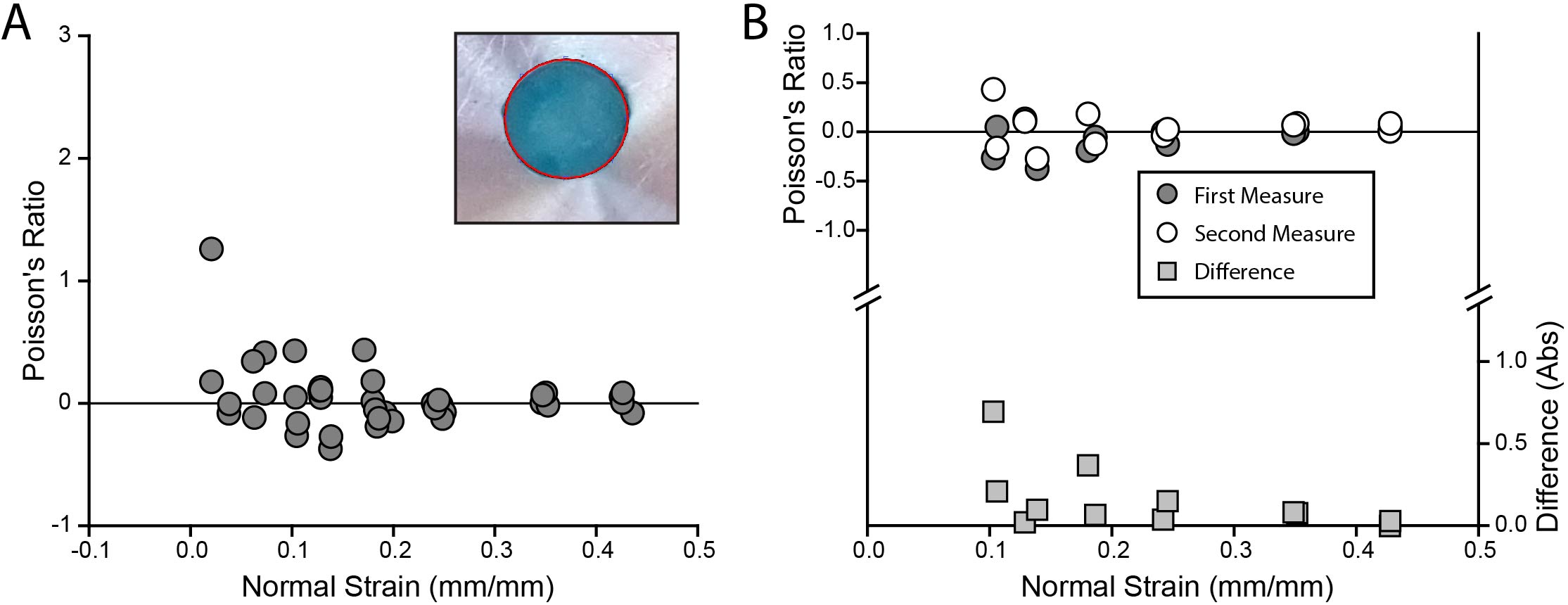


**Figure S7.8** Poisson’s ratio of FiHy™ networks. A) Poisson’s ratio is calculated for 0.25, 1, and 5 N compressive loads. *N* ≥ 2 per composition. B) The repeatability of Poisson’s ratio is calculated for 4 different samples. The absolute difference between the first and second measure demonstrates that below normal strains of ~0.25 mm/mm there is poor repeatability. At higher strains, Poisson’s ratio is approximately = 0. The y-axis is truncated to create separation between the plotted data.


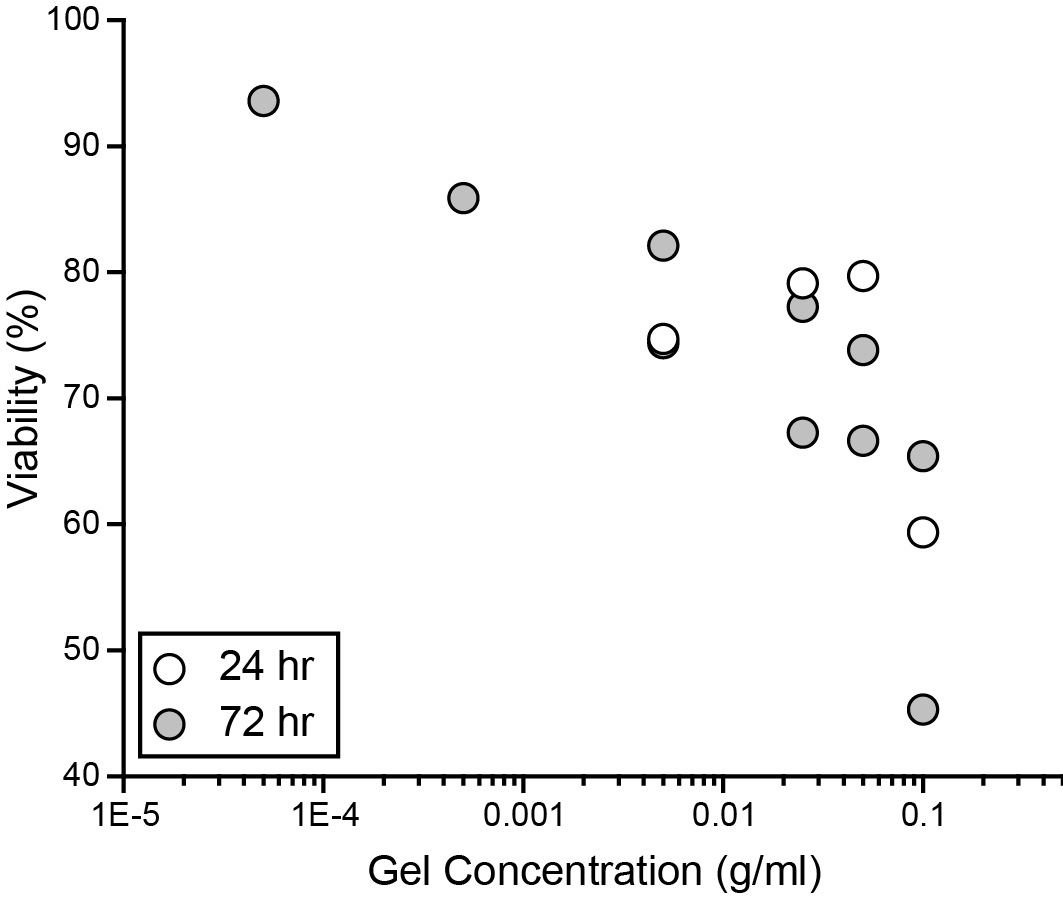


**Figure S7.9** alamarBlue^TM^ viability results for 24 and 72 hr of incubation with solubilized Gel. Comparison with the LIVE/DEAD^TM^ imaging suggest the Gel has a metabolic rather than cytotoxic effect. If we approximate the Gel concentration of the 3:1 FiHy™ scaffold as 70 wt% (**Figure 3**) and apply our media extraction conditions (0.2 g/ml) we get an estimated Gel concentration of 0.14 g/ml which takes us into the ‘cytotoxic’ range based on the alamarBlue™ assay.


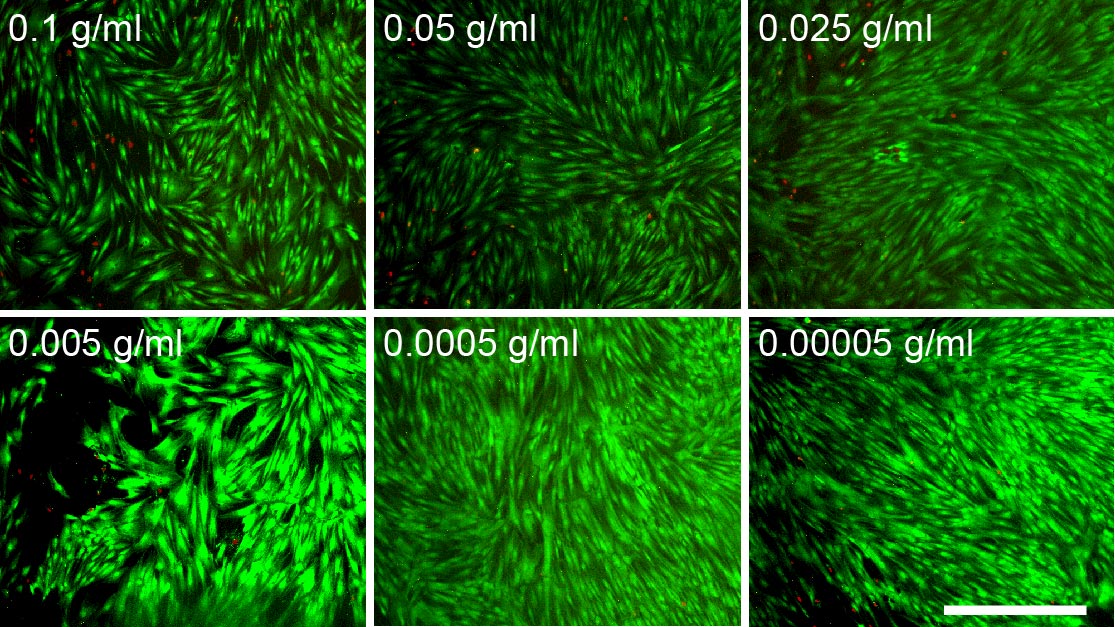


**Figure S7.10** Representative LIVE/DEAD™ images for six different solubilized Gel concentrations in hMSC media. Images were taken after 72 hr of extract exposure. Scale bar = 500 μm.

**
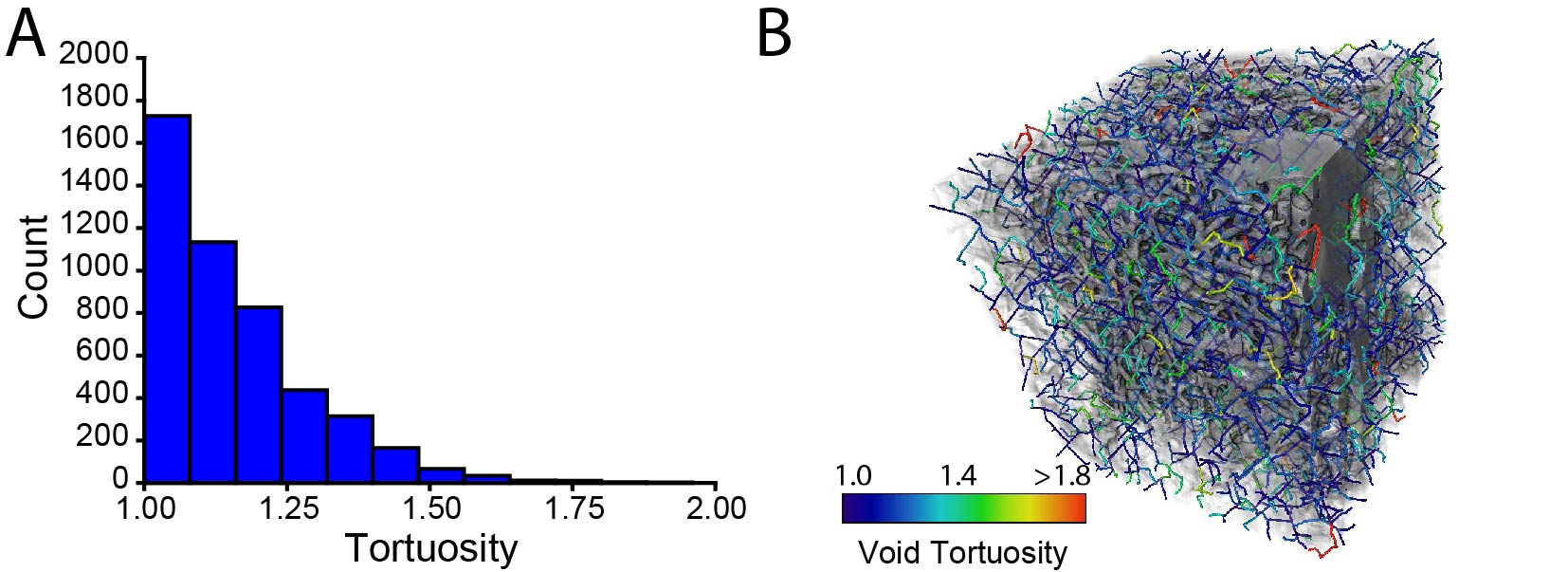
**

**Figure S7.11** A) The tortuosity of the pore space was quantified for a representative scaffold. B) 3D reconstruction of the pore space tortuosity with a partially eroded solid matrix (grey) to visualize the interconnected microporosity. Tortuosity is a measure of the path length relative to the end-to-end distance. Under this definition a straight line = 1 while any curvature yields a tortuosity > 1. The mean tortuosity was found to be 1.16 while 22.3% of pore spaces had a tortuosity greater than 1.25. Note that the sample was scanned under dry conditions; therefore, the tortuosity measured here is the microporosity of the dry state and will differ under hydrated conditions.

**
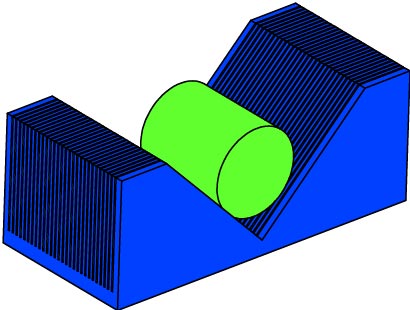
**

**Figure S7.12** V-block cutting guide to create a right cylinder of bovine articular cartilage (green cylinder). The fingers of the cutting guide are sized to fit standard microtome blades.


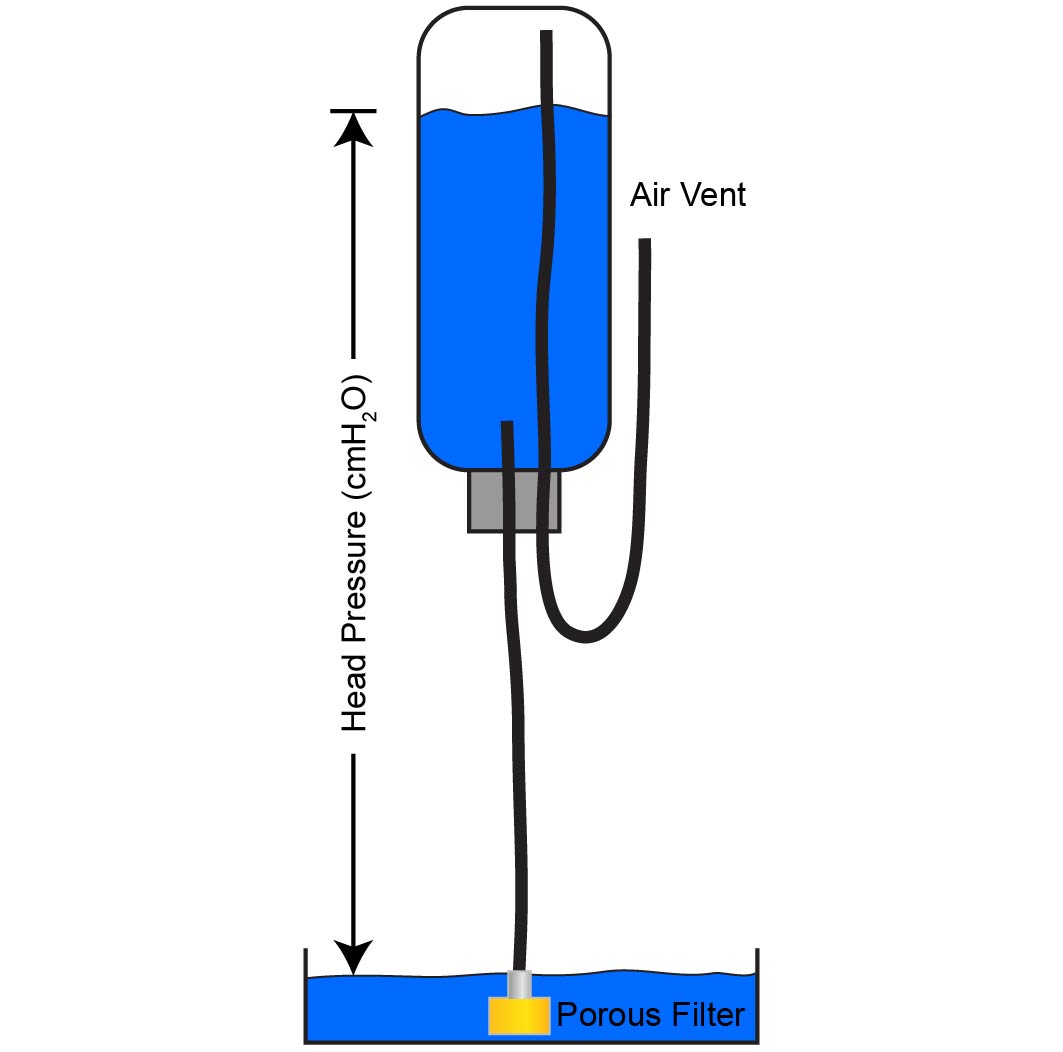


**Figure S7.13** Direct permeation experiment to determine the permeability of the porous filter relative to FiHy™ scaffolds.


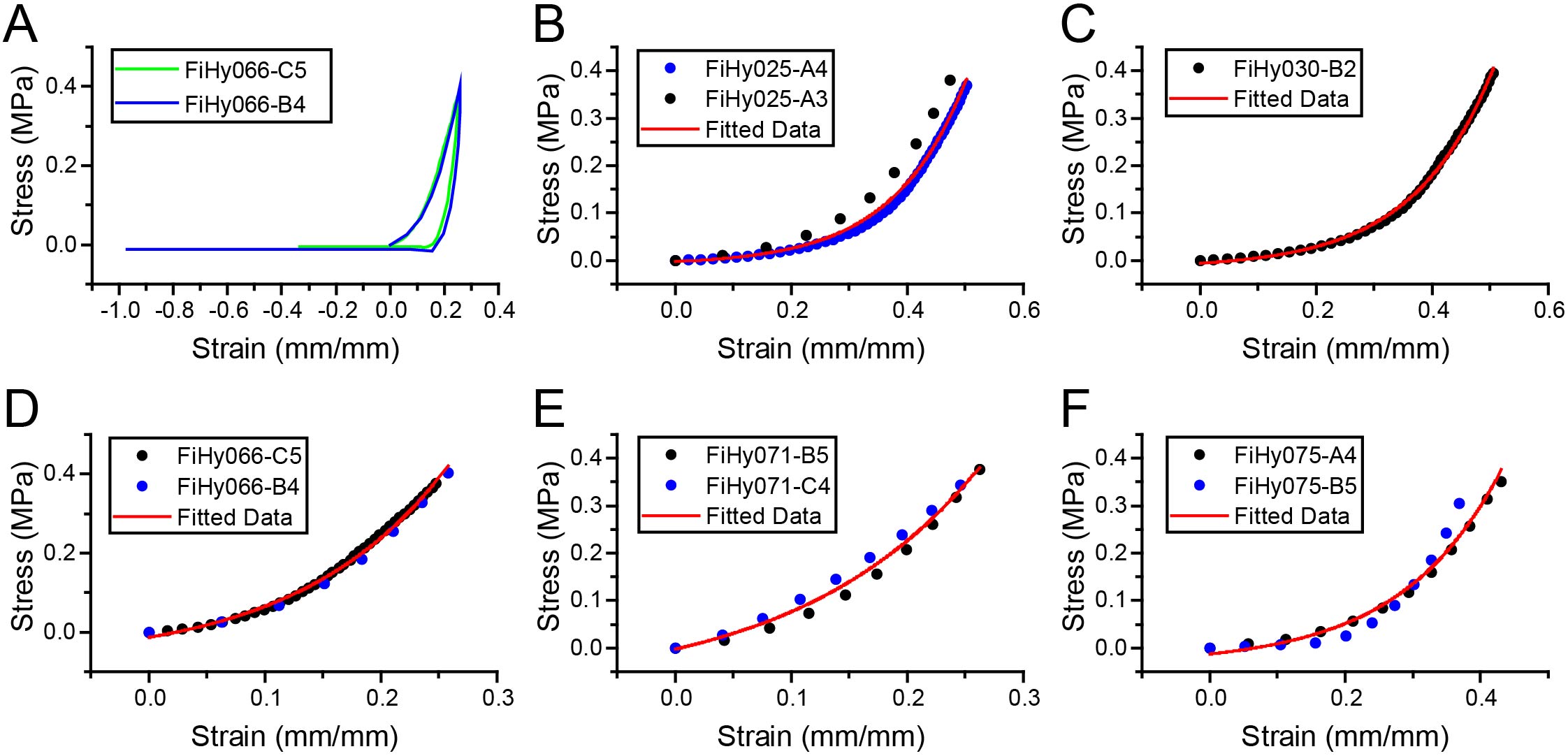


**Figure S7.14** Exponential model fits (red line) to unconfined compression stress-strain data for different FiHy™ scaffolds. The fit was used to estimate the normal strain for different compressive loads.


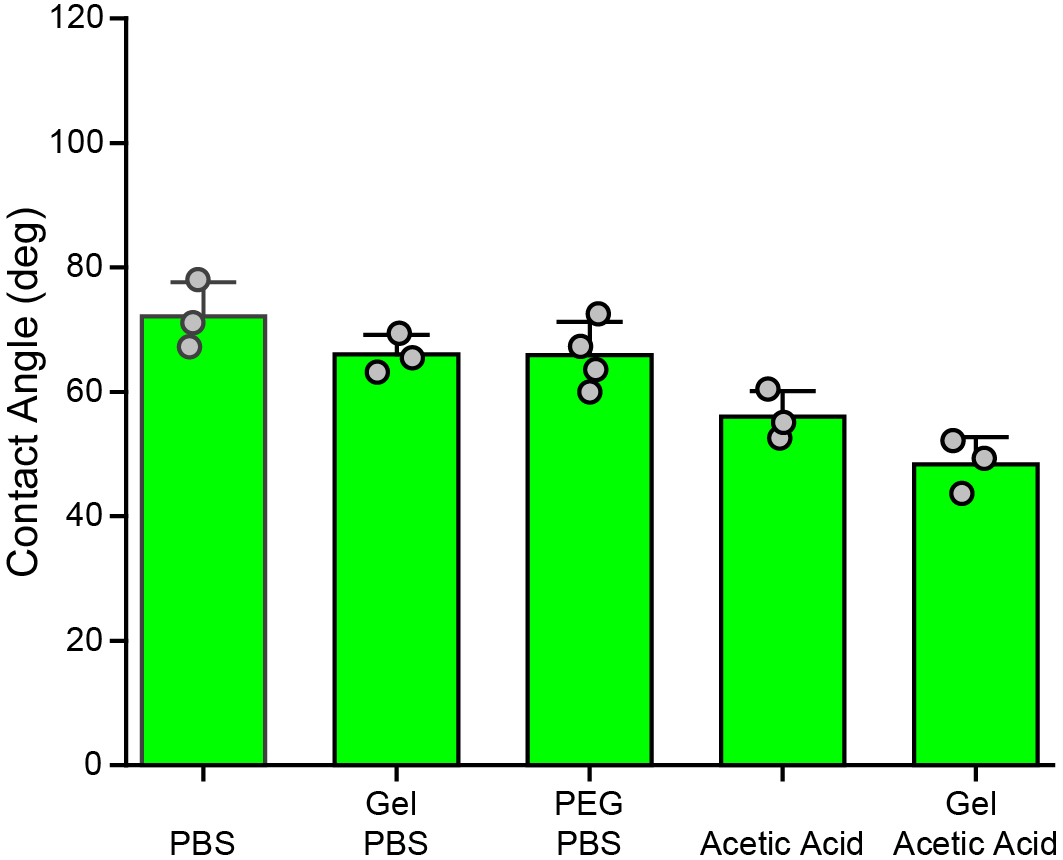


**Figure S7.15** Contact angle for different solvent (PBS and acetic acid) – polymer (Gel and PEG) systems on a PCL substrate. The Gel + acetic acid solution had significantly better wetting of PCL than PBS, acetic acid, Gel + PBS, and PEG + PBS. Significant differences were detected using a One-Way ANOVA with a Tukey test for multiple comparisons.

**Table S7.1** Electrospinning and electrospraying parameters.

| Settings | PCL emitter | Gel emitter | Drum |
| --- | --- | --- | --- |
| Voltage (kV) | 12 | 18 | -1 |
| Tip-to-Target (cm) | 12 | 6 | - |
| Flow Rate (ml/hr) | 2 | 2 | - |
| Emitter Gauge | 18 | 18 | - |
| Syringe (ml) | 20 | 20 or 50 | - |
| Collector Surface | - | - | Aluminum Foil |
| Collector Speed | - | - | 60 RPM, 200 mm/s |

**Table S7.2** Peak Fluid Load Fraction for different FiHy™ compositions and layers under 5 N loading (~0.2 MPa compressive stress). Mean ± 95% confidence interval, *N* ≥ 3. See **Figure 4** for a graphical representation of the data. For a 1:1 ratio, a One-Way ANOVA detected significant effects from layering (p<0.029). For 50 and 100 layers, a Two-Way ANOVA detected significant effects for Gel:PCL ratio (p<0.0001), but not layering (p<0.24).

| Layers | Gel:PCL Ratio | | | | |
| --- | --- | --- | --- | --- | --- |
|  | 0:1 | 1:1 | 2:1 | 3:1 | 1:0 |
| 2 | - | 0.27 ± 0.06 | - | - | - |
| 10 | - | 0.18 ± 0.02 | - | - | - |
| 50 | - | 0.18 ± 0.03 | 0.54 ± 0.14 | 0.68 ± 0.04 | - |
| 100 | - | 0.24 ± 0.20 | 0.49 ± 0.27 | 0.53 ± 0.15 | - |
| ∞ | 0.12 ± 0.07 | - | - | - | - |

**Table S7.3** Peak Fluid Load Fraction for different FiHy™ compositions and layers under 10 N loading (~0.4 MPa compressive stress). Mean ± 95% confidence interval, *N* ≥ 3. See **Figure 4** for a graphical representation of the data.

| Layers | Gel:PCL Ratio | | | | |
| --- | --- | --- | --- | --- | --- |
|  | 0:1 | 1:1 | 2:1 | 3:1 | 1:0 |
| 2 | - | 0.20 ± 0.08 | - | - | - |
| 10 | - | 0.12 ± 0.04 | - | - | - |
| 50 | - | 0.32 ± 0.13 | 0.53 ± 0.07 | 0.50 ± 0.04 | - |
| 100 | - | 0.29 ± 0.23 | 0.49 ± 0.24 | 0.36 ± 0.14 | - |
| ∞ | 0.11 ± 0.06 | - | - | - | - |

**Table S7.4** Nominal sample sizes of 6 and 10 mm are compared under unconfined creep compression at 5 and 10 N respectively. The permeability (k), equilibrium compressive modulus (E_S_), and effective tensile modulus (E_T_) are determined by fitting the TC model. The time constant (𝜏) is taken as the time for fluid load fraction to decay by 63%.

| Diameter (mm) | Fluid Load Fraction | k (/(N·s)) | E_S_ (MPa) | | E_T_ (MPa) | 𝜏 (s) |
| --- | --- | --- | --- | --- | --- | --- |
| 5.72 | 0.71 | 0.069 | | 0.29 | 1.40 | 115 |
| 10.33 | 0.80 | 0.023 | | 0.20 | 1.61 | 1020 |

**Table S7.5** Poroelastic model parameters for **Figures 7A** and **7B**. The respective model was fit to the 10 N loading condition. The fit parameters were then applied to every other condition to predict the creep response. Note the additional terms: *ν_θ_* (angular Poisson’s ratio), *ν_rz_* (rz Poisson ratio), and *φ_f_* (fluid fraction). To simplify the table, we set E_S_ = E_z_ and E_T­_ = E_r_. *For the NL model the permeability value is given for the unstressed reference condition *k_0_* and nonlinear coefficients *a* and *b*. (-) indicates that a material property is irrelevant to the model.

| Poroelastic Model | ν_θ_ | ν_rz_ | φ_f_ | k (mm^4^/(N·s)) | a | b | E_z_ (MPa) | E_r_ (MPa) |
| --- | --- | --- | --- | --- | --- | --- | --- | --- |
| TC | - | 0.0 | - | 0.023 | - | - | 0.20 | 1.61 |
| NL | 0.3 | 0.0 | 0.8 | 0.20* | 3 | 5 | 0.05 | 0.97 |

**Table S7.6** The NL and TC models were fit to a single bovine cartilage creep data set (**Figure 7C**). Note the additional terms: *ν_θ_* (angular Poisson’s ratio), *ν_rz_* (rz Poisson ratio), and *φ_f_* (fluid fraction). To simplify the table, we set E_S_ = E_z_ and E_T­_ = E_r_. *For the NL model the permeability value is given for the unstressed reference condition *k_0_* and nonlinear coefficients *a* and *b*. (-) indicates that a material property is irrelevant to the model.

| Poroelastic Model | ν_θ_ | ν_rz_ | φ_f_ | k (mm^4^/(N·s)) | a | b | E_z_ (MPa) | E_r_ (MPa) |
| --- | --- | --- | --- | --- | --- | --- | --- | --- |
| TC | - | 0.0 | - | 0.0006 | - | - | 1.04 | 10.4 |
| NL | 0.3 | 0.0 | 0.8 | 0.0015* | 3 | 3 | 0.43 | 7.33 |

**7.3** **Tension-Compression Poroelastic Model for Creep Unconfined Compression**

Mixture theory states that the total stress (σ_T_) on the system is the sum of the fluid (P) and solid (σ_S_) stresses [8].

$\sigma_{T}=P+\sigma_{S}$ (7.3.1)

σ_T_ is directly quantified as the applied load divided by the original cross-sectional area. To simplify the problem, the mixture stresses are decoupled and solved independently. Assuming linear elasticity and small deformations, Hooke’s Law can be used to calculate σ_S_ and solid force F_S._

$\sigma_{S}=E_{S}\cdot\varepsilon$ (7.3.2)

$F_{S}=E_{S}\cdot\pi\cdot R^{2}\cdot\varepsilon$ (7.3.3)

E_S_ is the compressive modulus of the solid matrix, ε is the engineering strain (1-h/h_0_), h is the cylinder height, and h_0_ is the unstressed cylinder height (**Figure S7.16**).


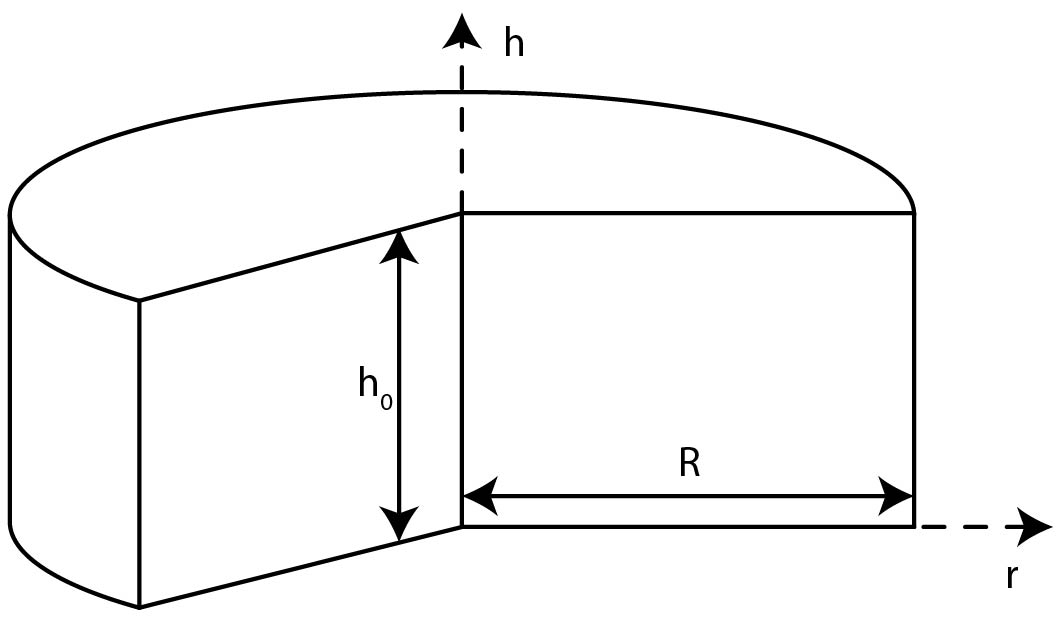


**Figure S7.16** Schematic of the analytical poroelastic model. The cylinder height (h), radius (r), and unstressed reference height (h_0_) and radius (R) are shown.

The fluid pressure is calculated using Darcy’s Law with the assumptions of isotropic and constant permeability. The following derivation is taken from the work of McCutchen [1].

Darcy’s Law relates a change in fluid pressure (P) to fluid velocity (V), path length (r), permeability (k), and fluid viscosity. We make no assumption about the fluid viscosity and instead absorb it into the permeability term with units of mm^4^/(N∙s), which is typical of articular cartilage mechanics studies.

$dP=\frac{V\cdot dr}{k}$ (7.3.4)

To determine the flow path, we assume a porous cylinder is deformed between two impermeable planes. We make the assumptions that fluid can only flow out of the cylinder’s radial surface and that a cylinder remains a cylinder during deformation. Under these conditions, the change in volume and volumetric flow (Q) are calculated as:

$\text{Change in Volume}=\pi\cdot R^{2}\cdot dh$ (7.3.5)

$Q=\pi\cdot R^{2}\left( \frac{dh}{dt} \right)=\pi\cdot R^{2}\cdot\dot{h}$ (7.3.6)

where R is the radius of the undeformed cylinder, dh is the axial deformation of the cylinder and $\dot{h}$ is the deformation rate of the cylinder. This change in volume must now be accounted for by either compressing the solid or fluid phase, expanding the cylinder radially, or exuding fluid from the cylinder. It has been shown that under physiological pressures the solid and fluid phases are virtually incompressible. In addition, articular cartilage and the composite structure described in this work have been shown experimentally to have a drained Poisson’s ratio of ~0; therefore we apply fluid exudation.

Since fluid must flow out radially, the radial surface area is calculated as:

$\text{A}_{\text{Radial}}=2\cdot\pi\cdot R\cdot h$ (7.3.7)

The fluid velocity is Q/A_Radial_:

$V=\frac{\pi\cdot R^{2}\cdot\dot{h}}{2\cdot\pi\cdot R\cdot h}=\frac{R\cdot\dot{h}}{2\cdot h}$ (7.3.8)

To generalize these equations to any radial position in the cylinder we replace R with r. **Equation 7.3.8** can now be substituted into **Equation 7.3.4**:

$dP=\frac{r\cdot\dot{h}}{2\cdot h}\cdot\frac{dr}{k}=\frac{r}{2\cdot k}\cdot\frac{\dot{h}}{h}\cdot dr$ (7.3.9)

which upon integration gives the radial fluid pressure (P(r)), and by integrating over the differential annuli gives the fluid force (F_P_).

$P\left( r \right)=\frac{1}{4\cdot k}\cdot\frac{\dot{h}}{h}(R^{2}-r^{2})$ (7.3.10)

$F_{P}=\frac{\pi\cdot R^{4}}{8\cdot k}\cdot\frac{\dot{h}}{h}$ (7.3.11)

The solid stress (σ_S_) and solid force (F_S_) are calculated using linear elasticity.

$$\sigma_{S}=E_{S}\left( 1-\frac{h}{h_{0}} \right)$$

$F_{S}=E_{S}\cdot\pi\cdot R^{2}\left( 1-\frac{h}{h_{0}} \right)$ (7.3.12)

where E_S_ is the equilibrium compressive modulus of the solid matrix. The total load (F_T_) supported by the poroelastic material is then:

$$F_{T}=F_{P}+F_{S}$$

$F_{T}=-\frac{\pi\cdot R^{4}}{8\cdot k}\cdot\frac{\dot{h}}{h}+E_{S}\cdot\pi\cdot R^{2}\left( 1-\frac{h}{h_{0}} \right)$ (7.3.13)

By convention fluid pressure is negative hence the negative sign at the front of **Equation 7.3.13**. Before solving the differential equation, we will establish the height immediately after the application of load. We make the assumption that loading is instantaneous and that no fluid exudation has occurred. Under a small strain assumption, the radial strains are ½ the normal strains. Therefore, fluid pressure is limited by a radial strain that is 0.5 the normal strain (ɛ_N_).

$P=E_{S}\cdot\varepsilon_{N}\cdot0.5=0.5\cdot\sigma_{S}$

$F_{P}=0.5\cdot F_{S}$ (7.3.14)

However, if the solid modulus is different in tension (E_T_), then P can exceed 0.5 σ_S_. We include the tension compression nonlinearity to account for the effect of fiber reinforcement.

$P=E_{T}\cdot\varepsilon_{N}\cdot0.5$ (7.3.15)

$$E^{*}=\frac{E_{T}}{E_{S}}$$

The height immediately after the application of load (h_i_) becomes:

$h_{i}=h_{0}-\frac{\sigma_{T}}{{E^{*}\cdot E_{S}\cdot0.5+E}_{S}}h_{0}$ (7.3.16)

Now we can solve the differential **Equation 7.3.17** using 7.3.16 as an initial condition.

$$F_{T}=A\frac{-\dot{h}\left( t \right)}{h\left( t \right)}+B\left( 1-\frac{h\left( t \right)}{h_{0}} \right)$$

$A=\frac{\pi\cdot R^{4}}{8\cdot k};B=E_{S}\cdot R^{2}\cdot\pi$ (7.3.17)

The solution is:

$$h\left( t \right)=\frac{-h_{0}\left( B-F_{T} \right)e^{\frac{B}{A}\cdot t+B\cdot h_{0}*C}}{e^{\frac{F_{T}}{A}\cdot t+F_{T}\cdot h_{0}\cdot C}-B\cdot e^{\frac{B}{A}\cdot t+B\cdot h_{0}*C}}$$

$C=\frac{\ln\left( -{h_{i}}/{(B\cdot}h_{0}-B\cdot h_{i}-F_{T}\cdot h_{0} \right))}{\left( -F_{T}+B \right)h_{0}}$ (7.3.18)

The expression for $\dot{h}(t)$ can be found by taking the derivative:

$\dot{h}\left( t \right)=\frac{M\left( R-P \right)\cdot e^{N+P\cdot t+Q+R\cdot t}}{\left( B\cdot e^{N+R\cdot t}-e^{P\cdot t+Q} \right)^{2}}$ (7.3.19)

$$M=-h_{0}\left( B-F_{T} \right)$$

$$N=B\cdot h_{0}\cdot C$$

$$P=F_{T}/M$$

$$Q=F_{T}\cdot h_{0}\cdot C$$

$$R=B/M$$

Based on mixture theory the fluid load fraction (F’) is the fraction of the applied load (F_T_) supported by F_P_:

$F^{'}=\frac{F_{P}}{F_{P}+F_{S}}=\frac{F_{P}}{F_{T}}$

$F^{'}=\frac{-A\frac{\dot{h}}{h}}{-A\frac{\dot{h}}{h}+B\left( 1-\frac{h}{h_{0}} \right)}$ (7.3.20)

**Equations 7.3.18** and **7.3.19** can now be substituted into **Equation 7.3.20** to give the fluid load fraction as a function of time, geometry (R, h_0_), and material properties (E_S_, E*, k).

**7.4** **Transversely Isotropic Poroelastic Model for Creep Unconfined Compression**

The key assumption of the nonlinear poroelastic model is that the sample remains cylindrical during compression, resulting in a time-dependent sample height $h\left( t \right)$ and radius $R\left( t \right)$, with $h\left( 0 \right)=h_{0}$ and $R\left( 0 \right)=R_{0}$. The radial and axial coordinates are written as $r$ and $z$. Following MacMinn *et al.* [47], the model is posed in an Eulerian coordinate system. The principal stretches of the sample along the radial, orthoradial, and axial directions are given by $\lambda_{r}\left( r,t \right)$, $\lambda_{\theta}\left( r,t \right)$, and $\lambda_{z}\left( t \right)$, respectively. The height of the sample is related to the axial stretch via $h=\lambda_{z}h_{0}$, which, in turn, allows the mean axial strain to be defined as $\epsilon=\left( h-h_{0} \right)/h_{0}=\lambda_{z}-1$. The radial and axial stretches can be written in terms of the radial displacement $u_{s}\left( r,t \right)$ as:

$\lambda_{r}=\left( 1-\frac{\partial u_{s}}{\partial r} \right)^{-1}, \lambda_{\theta}=\left( 1-\frac{u_{s}}{r} \right)^{-1}$ (7.4.1)

The mechanical response of the sample is described using Hencky (or logarithmic) strains given by $\varepsilon_{i}=\log\lambda_{i}$ along with a transversely isotropic stress-strain relation [10]:

$\left( \begin{matrix} \sigma_{r} \\ \sigma_{\theta} \\ \sigma_{z} \end{matrix} \right)=\frac{1}{J}\left( \begin{matrix} C_{11} & C_{12} & C_{13} \\ C_{12} & C_{11} & C_{13} \\ C_{13} & C_{13} & C_{33} \end{matrix} \right)\left( \begin{matrix} \varepsilon_{r} \\ \varepsilon_{\theta} \\ \varepsilon_{z} \end{matrix} \right)$ (7.4.2)

where $J=\lambda_{r}\lambda_{\theta}\lambda_{z}$ and $\sigma_{i}$ are the principal elastic stresses. The stiffness parameters $C_{ij}$ can be related to the radial and axial Young's moduli, $E_{r}$ and $E_{z}$, as well as the in-plane and out-of-plane Poisson's ratios $\nu_{r\theta}$ and $\nu_{zr}=\nu_{rz}E_{z}/E_{r}$ as:

$C_{11}=E_{r}\left( 1-\nu_{zr}^{2}E_{r}/E_{z} \right)/\left[ \left( 1+\nu_{r\theta} \right)\Delta\right]$ (7.4.3)

$C_{12}=E_{r}\left( \nu_{r\theta}+\nu_{zr}^{2}E_{r}/E_{z} \right)/\left[ \left( 1+\nu_{r\theta} \right)\Delta\right]$ (7.4.4)

$C_{13}=E_{r}\nu_{zr}/\Delta$ (7.4.5$)$

$C_{33}=E_{z}\left[ 1+2\nu_{zr}^{2}\left( E_{r}/E_{z} \right)/\Delta\right]$ (7.4.6)

where $\Delta=1-\nu_{r\theta}-2\nu_{zr}^{2}E_{r}/E_{z}$.

The radial stress balance is given by:

$\frac{\partial\sigma_{r}}{\partial r}+\frac{\sigma_{r}-\sigma_{\theta}}{r}=\frac{\partial p}{\partial r}$ (7.4.7)

where $p\left( r,t \right)$ is the fluid pressure. The transport of fluid through the porous solid is described by Darcy's law, which for this model can be written as:

$\lambda_{r}\frac{\partial u_{s}}{\partial t}+\frac{r}{2}\frac{\dot{\lambda_{z}}}{\lambda_{z}}=\frac{k\left( \phi_{f} \right)}{\mu}\frac{\partial p}{\partial r}$ (7.4.8)

where $\dot{\lambda_{z}}=d\lambda_{z}/dt$ and $\mu_{f}$ is the kinematic viscosity of the fluid. The dependence of the permeability $k$ on the porosity $\phi_{f}$ is captured through a generalised Kozeny-Carmen law of the form:

$\frac{k\left( \phi_{f} \right)}{k_{0}}=\frac{\left( 1-\phi_{f,0} \right)^{b}}{\phi_{f,0}^{a}}\frac{\phi_{f}^{a}}{\left( 1-\phi_{f} \right)^{b}}$ (7.4.9)

where $k_{0}=k\left( \phi_{f,0} \right)$ is the initial permeability of a sample with initial porosity $\phi_{f,0}$. The quantities $a$ and $b$ are fitting parameters, which are $a = 3$ and $b = 2$ in the classical Kozeny-Carman law. The sample is assumed to be incompressible, which means that volumetric changes in material elements can only occur due to a gain or loss of fluid. This is expressed through the condition:

$\lambda_{r}\lambda_{\theta}\lambda_{z}=\frac{1-\phi_{f,0}}{1-\phi_{f}}$ (7.4.10)

**Equation 7.4.10** can be used to solve for the porosity, which must be a function of only the radial coordinate and time, $\phi_{f}=\phi_{f}\left( r,t \right)$. This model constitutes a one-dimensional system of partial differential equations that must be solved in $r$ and $t$ with the boundary conditions:

$$u_{s}\left( 0,t \right)=0; \sigma_{r}\left( R\left( t \right),t \right)=0; p\left( R\left( t \right),t \right)=0$$

where $R$ is determined from the equation $u_{s}\left( R\left( t \right),t \right)=R\left( t \right)-R_{0}$. The axial stretch $\lambda_{z}$ is determined from the force balance:

$F=2\pi\int_{0}^{R\left( t \right)} \left( \sigma_{z}-p \right)r dr$ (7.4.11)

where $F$ is the load imposed on the sample. The initial conditions that are used when numerically simulating the model will be discussed in the next section.

**Instantaneous response**

The instantaneous response of the sample corresponds to a sudden axial compression and radial expansion with spatially uniform stretches given by $\lambda_{r}=\lambda_{\theta}=\lambda_{z}^{-1/2}$. The axial stretch can be obtained by numerically solving the force balance:

$\frac{F}{\pi R_{0}^{2}}=\left( \frac{\left( 1-2\nu_{zr} \right)^{2}E_{r}}{2\Delta}+E_{z} \right)\frac{\log\lambda_{z}}{\lambda_{z}}$ (7.4.12)

The radius of the sample is $R=\lambda_{z}^{-1/2}R_{0}$ and the radial displacement is given by $u_{s}=r\left( 1-\sqrt{\lambda_{z}} \right)$. The uniform fluid pressure in the sample can be expressed as:

$p=-\left[ \frac{\left( 1-2\nu_{zr} \right)E_{r}}{2\Delta} \right]\log\lambda_{z}$ (7.4.13)

Note that $p > 0$ since $\lambda_{z}<1$. The solutions describing the instantaneous response are used as initial conditions when numerically simulating the full nonlinear poroelastic model.

**Equilibrium response**

The equilibrium state is calculated by setting the fluid pressure to zero, $p = 0$, and looking for time-independent homogeneous stretches with $\lambda_{\theta}=\lambda_{r}$. The radial and axial stretches are related by $\log\lambda_{r}=-\nu_{zr}\log\lambda_{z}$. The force balance can be evaluated to give:

$\frac{F}{\pi R_{0}^{2}E_{z}}=\frac{\log\lambda_{z}}{\lambda_{z}}$ (7.4.14)

**Fitting procedure**

By treating the Poisson’s ratios $\nu_{zr}$ and $\nu_{r\theta}$ as known parameters and using the experimental data to determine the instantaneous and equilibrium values of $\lambda_{z}$, the Young’s moduli $E_{r}$ and $E_{z}$ can be computed using **Equations 7.4.12** and **7.4.14**. The initial permeability $k_{0}$ can be eliminated from the model by introducing a non-dimensional time $t’=t/\tau_{p}$, where $\tau_{p}=\left( R_{0}^{2} \right)/\left( k_{0}C_{11} \right)$ is the poroelastic time scale. Given values of $a$ and $b$, the model is numerically solved once using a hand-coded implicit finite-difference scheme in MATLAB^®^ 2020A. The mean axial strain $\epsilon$ is then normalized using its minimum and maximum values, $\epsilon_{min}$ and $\epsilon_{max}$, according to:

$\epsilon_{n}=\frac{\epsilon-\epsilon_{min}}{\epsilon_{max}-\epsilon_{min}}$ (7.4.15)

The times $t_{n}$ at which $\epsilon_{n}=0.25$, $0.50$, and $0.75$ are determined from the experimental data. Similarly, the corresponding non-dimensional times $t_{n}’$ are then computed from the model. The poroelastic time scale $\tau_{p}$ is found by minimizing the least-square error between the experimental values of $t_{n}$ and those predicted from the model, $\tau_{p}t_{n}’$. Once $\tau_{p}$ is known, the permeability $k_{0}$ can be computed.
